# Management of late urinary side effects after pelvic radiation therapy: a scoping review

**DOI:** 10.1016/j.ctro.2026.101332

**Published:** 2026-10-21

**Authors:** Laura Mullaney, Salma Bennani

**Affiliations:** Applied Radiation Therapy Trinity (ARTT), Discipline of Radiation Therapy, School of Medicine, Trinity St. James's Cancer Institute, Trinity College Dublin, University of Dublin, Dublin, Ireland

**Keywords:** Pelvic radiation therapy, Late urinary side effects, Late urinary toxicity, Radiation cystitis, Urethral stricture, Urinary diversion, Patient-reported outcomes

## Abstract

**Background:**

Radiation therapy (RT) is a cornerstone treatment for pelvic malignancies but can cause durable urinary side effects that impair quality of life. Presentations range from haematuria and irritative symptoms to stricture, fistula, osteomyelitis, and end-stage urinary failure. This scoping review maps the management strategies for late urinary side effects after pelvic RT.

**Methods:**

A PRISMA-ScR-guided search of PubMed and Embase identified studies published from January 2015 to April 2026. Primary studies reporting management outcomes were included. Two reviewers assessed full-text eligibility. Data were charted by syndrome, intervention, design, population, RT characteristics, follow-up and outcomes. Risk of bias in randomised trials was assessed using RoB.

**Results:**

69 studies of previously irradiated patients were included, predominantly retrospective cohorts. RT modality was reported in 56% of publications, total dose in 33%, and the interval from RT to toxicity in 70%. Hyperbaric oxygen therapy (HBOT) had the strongest evidence for radiation cystitis, supported by randomised data and long-term follow-up. Reconstructive surgery for urethral and ureteral strictures achieved acceptable patency, but recurrence and incontinence remained common. Fistula, osteomyelitis, and refractory bleeding were often managed with salvage diversion in severe or refractory cases, which reduced pain and treatment burden despite substantial perioperative morbidity.

**Conclusions:**

Evidence is strongest for HBOT in chronic radiation cystitis. Elsewhere, management rests on retrospective, expert-centre series. Clinical decision-making is best guided by syndrome, severity, and tissue quality, treating prior RT modality and dose as markers of injury burden, with timely referral and shared decision-making when repeated intervention fails to provide durable control.

## Highlights

• 69 studies map the management of late urinary side effects after pelvic radiation therapy.
• Hyperbaric oxygen has the strongest randomised, long-term evidence for cystitis.
• Stricture repair achieves patency, but recurrence and incontinence remain common.
• Salvage diversion reduces pain, transfusions and admissions despite high morbidity.
• Evidence supports timely referral and shared decision-making on salvage surgery.

## Introduction

Radiation therapy (RT) is administered to approximately half of all patients with pelvic malignancies, including those with prostate, colorectal, and gynaecological cancers, and constitutes a cornerstone of both curative and adjuvant treatment in this setting [1], [2], [3]. Advances in treatment delivery, including intensity-modulated RT (IMRT) and image-guided RT (IGRT), have improved tumour targeting and reduced dose exposure to normal tissue [4], [5]. Nevertheless, the anatomical proximity of the urinary tract to pelvic radiation fields means that late urinary side effects remain an important and persistent clinical consequence of treatment [6], [7].

Late urinary side effects after pelvic RT represent a broad and clinically heterogeneous spectrum of injury, ranging from haematuria and irritative lower urinary tract symptoms (LUTS) to urethral and ureteral stricture, fistula, pubic osteomyelitis resulting from chronic urosymphyseal fistula and pelvic sepsis, to end-stage urinary injury [6], [8], [9]. These side effects typically emerge months to years after radiation exposure, driven by microvascular injury, progressive fibrosis, and chronic inflammatory change that impair healing and increase susceptibility to erosion, infection, and stenosis [6], [7]. Reported incidence varies widely by cancer site and grading system, with Grade 2 or higher genitourinary side effects (scored by Radiation Therapy Oncology Group/European Organisation for Research and Treatment of Cancer (RTOG/EORTC) late radiation morbidity criteria or Common Terminology Criteria for Adverse Events (CTCAE)) affecting approximately 12% to 27% of patients [10], [11], [12], [13], [14], [15].

The consequences of late urinary side effects extend beyond anatomical injury, contributing to chronic sleep disruption, social and occupational withdrawal, and substantial, lasting decrease in quality of life (QoL) [6], [16], [17]. Management is further complicated by radiation altering the meaning of procedural success itself: urethral patency may be restored at the cost of stress incontinence, haematuria may be controlled but recur, and salvage surgery, despite substantial morbidity, may still produce the most durable relief [18], [19], [20].

Despite this clinical significance, the evidence base is fragmented due to heterogeneous populations, variable outcome definitions, and inconsistent follow-up, and limited separate reporting of outcomes in irradiated patients within combined-modality cohorts [21]. Existing reviews address individual syndromes or interventions in isolation; to our knowledge, none has mapped the full spectrum of post-radiation urinary side effects across management pathways. For clinicians managing long-term survivors, this fragmentation leaves limited consolidated guidance on matching treatment strategies to injury patterns and severity.

A scoping review methodology was selected due to the high degree of heterogeneity in the literature. This scoping review maps the contemporary management literature for late urinary side effects after pelvic RT and aims to provide a clinician-facing overview of management strategies, the syndromes in which they are used, and the strengths and limitations of the supporting evidence to aid effective management for these patients.

## Materials and methods

This scoping review was conducted in accordance with the Preferred Reporting Items for Systematic Reviews and Meta-Analyses Extension for Scoping Reviews (PRISMA-ScR) guidelines [22]. The review question was framed using the Participants, Concept, and Context (PCC) framework. Inclusion and exclusion criteria are presented in Table 1. No review protocol was registered; PRISMA-ScR was used as the reporting framework.

**Table 1:** Participants, concept, and context of scoping review, with inclusion and exclusion criteria.

	Inclusion	Exclusion
Participants	Adult (≥18 years) male and female patients treated for pelvic malignancies with radiation therapy (RT)	Paediatric populations (age < 18 years); patients not treated with pelvic RT; studies of RT delivered for non-pelvic malignancies
Concept	Management interventions for late urinary side effects following pelvic RT; studies must report clinical outcomes of management interventions	Studies reporting only acute urinary side effects and side effect grading; dosimetric, planning, or prevention studies; narrative reviews, editorials, letters, conference abstracts, and individual case reports or case series of five or fewer patients
Context	Primary studies (randomised controlled trials, prospective cohort studies, and retrospective cohort studies) in which RT (EBRT, BT, or combined EBRT plus BT) was delivered to all patients in the reported cohort	Studies in which RT was not delivered to all patients, including mixed-treatment cohorts that do not separately report RT-specific outcomes; non-English language publications; studies published before January 1, 2015; articles where full text could not be sourced

Abbreviations: BT, brachytherapy; EBRT, external beam radiation therapy; RT, radiation therapy.

### Search strategy, sources, and screening

PubMed and Embase (Embase.com, Elsevier) were searched for eligible studies published from January 1, 2015 to April 29, 2026. PubMed and Embase were selected as complementary core biomedical databases: PubMed for MEDLINE-indexed clinical literature and Embase for its broader indexing of European journals. No supplementary search methods were used. This start date was selected to capture contemporary management practice and was applied pragmatically to bound this review. The search combined terms related to pelvic RT, urinary side effects, and management. The full search strategy is provided in Appendix A. Duplicates were removed, and screening was conducted in Covidence (Veritas Health Innovation, Melbourne, Australia). Titles and abstracts were screened against the eligibility criteria by a single reviewer (LM). All records advancing to full-text assessment were then assessed against the eligibility criteria by two reviewers (LM, SB). Agreement between reviewers on full-text eligibility was 90.8% (Cohen's κ = 0.80), and the discordant decisions were resolved by consensus.

Studies reporting cohorts of five or fewer patients were excluded, as such series function as case reports and provide insufficient data to inform a management framework. Eligibility was restricted to cohorts in which all patients had received pelvic RT. Surgery and chemoradiation independently cause urinary injury, so mixed-treatment cohorts without separate reporting for irradiated patients preclude attribution of injury patterns or responses to radiation, and management outcomes in irradiated tissue are not directly transferable to non-irradiated populations. This review addresses chronic established late urinary side effects, defined by the RTOG/EORTC convention as arising more than 90 days after radiation therapy [23]. Eligibility required studies to report outcomes of a management intervention for such injury; timing of onset was not applied as a separate screening criterion, and the interval from RT to toxicity or its treatment was charted where reported.

All included records were screened for shared patient populations by pairwise comparison of author lists. Pairs sharing two or more authors were checked against institution and recruitment period, and corresponding authors were contacted where the relationship remained unclear. The unit of analysis was the study rather than the publication: publications reporting the same study were charted as one, and those reporting different analyses of overlapping populations were retained separately.

### Classification framework

For analytic consistency, each included study was assigned to a primary urinary syndrome category and a primary management pathway. These represent two independent classification dimensions: the syndrome category describes the dominant clinical condition being treated, and the management strategy describes the therapeutic intent of the intervention.

Urinary syndrome categories were defined a priori as follows: (1) radiation cystitis and haematuria; (2) irritative LUTS and urinary symptom-related bother; (3) stress urinary incontinence; (4) urethral stricture or outlet stenosis; (5) ureteral stricture or upper tract obstruction; (6) fistula, pubic osteomyelitis and (7) end-stage urinary injury (refractory bleeding or devastated outlet). Management strategies were defined as: (a) bladder-preserving, where the native bladder was retained and treatment aimed to control symptoms or bleeding without major reconstruction or diversion; (b) reconstructive, where the aim was to restore urinary tract patency, continuity, storage, or drainage; and (c) salvage, where treatment was directed at severe, refractory, or destructive disease, typically involving urinary diversion with or without cystectomy or other extirpative surgery.

Classification was performed at a study level. Where a study addressed more than one syndrome or management strategy, it was assigned to the category representing the dominant clinical problem and the highest-intensity intervention.

### Data extraction

A data extraction form was developed and piloted on three studies prior to full extraction. Data charted from each included study comprised: publication year, study design, sample size and sex distribution, cancer site, urinary syndrome category, management intervention, follow-up duration, outcome definitions, patient-reported outcome measures (PROMs) where available, side effects, and need for reintervention. Data were extracted by one reviewer (LM) and verified by a second reviewer (SB) for a 20% sample of included studies; one discrepancy, concerning the disease-site composition of a single cohort, was identified and corrected. Because interventions addressed different disease severities and syndromes across heterogeneous populations, formal meta-analysis was not attempted; results were synthesised narratively and mapped by syndrome category and management pathway.

### Risk of bias assessment

The two randomised trials were assessed using RoB 2 [24]. Non-randomised studies, predominantly uncontrolled case series, were not formally appraised; their design, sample size, and follow-up are reported in Table 3, Table 4, Table 5. Assessment was by a single reviewer (LM) and did not determine eligibility, inclusion, or weighting.

**Table 3:** Characteristics of studies of bladder-preserving management for late urinary side effects after pelvic RT.

Author, year	Study design; n, sex; disease site	RT characteristics	Intervention & management strategy	Follow-up	Primary objective	Main findings & key insight
Oscarsson 2019, 2025 [25], [26]	Randomised controlled trial (phase 2–3) with 5-year single-group follow-up; 87 randomised, 79 analysed, mixed; pelvic cancers, mainly prostate; 70 in long-term follow-up	EBRT mean 64 Gy; BT boost (arm means 21 and 18.2 Gy) in 29% and 34%; randomisation stratified by sex, time from RT (<12 vs ≥12 mo) and prior pelvic surgery; dose–response not assessed	HBOT versus standard care to 6 to 8 months; control patients then offered HBOT; single-group follow-up of all HBOT-treated patients	6 to 8 months(primary); 5 years (follow-up)	Test whether HBOT improves urinary outcomes in late radiation cystitis and assess durability of benefit	EPIC urinary total score improved by 10.1 points more than standard care, and 64% versus 18% improved cystoscopically. In uncontrolled follow-up of 70 HBOT-treated patients from both arms, mean EPIC urinary total score remained 19.1 points above baseline at 5 years; 68.6% were responders at 6 months after HBOT and 12.8% received additional HBOT. Key insight: the only randomised evidence for HBOT; five-year data are uncontrolled, and the open-label design leaves the patient-reported primary outcome vulnerable to bias
Allameh 2020 [27]	Randomised controlled trial; 60 randomised, 58 analysed, mixed; pelvic malignancies with radiation cystitis	Conventional or IMRT; 30–70 Gy by site (prostate 55–70 Gy/33–36 fr; cervix 50–60 Gy/25–28 fr; rectum 45–60 Gy; endometrium 45 Gy; ovary 30 Gy); enrolled ≥6 mo after RT	Intravesical sodium hyaluronate versus saline	9 months	Compare symptom and QoL benefit of sodium hyaluronate	Hyaluronate improved haematuria, pain, and King's Health Questionnaire domains versus saline. Key insight: RCT, supportive comparative evidence exists, but the trial is small and follow-up is limited.
Pereira 2020 [28]	Retrospective cohort; 105, mixed; radiation haemorrhagic cystitis, commonly after prostate or cervical RT	Modality NR; total dose available in 22% (median 70 Gy prostate, 50 Gy cervix); median 28 mo (1–240) RT to haematuria; median 8 mo haematuria to HBOT	HBOT, median 40 sessions	Median 64 months	Assess efficacy and predictors of response to HBOT	Haematuria control was achieved in 92.4%, with recurrence in 24.7%; dysuria, frequency, and haematuria scores also improved. Key insight: earlier treatment predicted better response and less recurrence.
Soriano 2026 [29]	Retrospective, two-site comparison; 93 (54 vs 39), mixed; RHC	EBRT 38% vs 10%, IMRT 19% vs 15%, SBRT 6% vs 37% (2.0 vs 2.5 ATA cohorts); dose NR; mean 6–7 y since cancer diagnosis	HBOT at 2.0 vs 2.5 ATA	1 year	Compare efficacy/safety of HBOT pressure protocols	2.5 ATA: lower 1-year haematuria recurrence (19.5% vs 38.5%, *p* = 0.018; confirmed *p* = 0.003 on propensity-adjusted analysis) with more adverse events on bootstrap analysis only (*p* < 0.001; not on univariate or propensity-adjusted analysis).
Milton 2025 [30]	Retrospective cohort with PROM follow-up; 89 treated, 54 patient-reported outcomes; predominantly male; radiation cystitis after external-beam RT, almost entirely prostate cancer	EBRT (all); dose NR; median 7 y (IQR 4–10) RT to HBOT	HBOT with long-term patient-reported follow-up	Follow-up after treatment completion, cohort treated from 2017 to 2023	Assess long-term patient-reported outcomes after HBOT for radiation cystitis	Mean symptom severity improved from 7.9 to 2.0 and anxiety from 6.9 to 2.1; general practitioner visits, emergency presentations, and transfusions also fell, although 39% still reported intermittent haematuria and 26% required further treatment. Key insight: HBOT can produce durable patient-perceived benefit even when some residual or recurrent bleeding persists.
Mougin 2016 [31]	Retrospective cohort; 71, mixed; pelvic cancers, mainly prostate	EBRT 91.5%, BT 3%, EBRT+BT 5.5%; median 66 Gy (45–138); median 38 mo (1–384) RT to haematuria	HBOT with additional sessions when needed	Median 15 months	Assess overall success and predictors in radiation haemorrhagic cystitis	Overall success was 64.8%, with recurrence in 26.8%. Key insight: lower baseline haematuria grade predicted better outcome.
de Angelis 2025 [32]	Retrospective cohort; 61, mixed (89% prostate); RIC with haematuria, ED presentations	Prostate RT: primary 32%, adjuvant 42%, salvage 26% (of 54 prostate patients); modality and dose NR; median 7 y (IQR 2–9) RT to first cystitis episode	Retrospective ED-management/recurrence analysis	Median RT-to-onset 7 years, follow-up 33 months	Identify predictors of recurrent ED visits/hospitalisation	52% hospitalised at first presentation; 75% had a further RIC episode during follow-up; age (HR 1.01) and anticoagulant/antiplatelet use (HR 1.8) independently predicted recurrence; 11% developed secondary bladder cancer; 11% required radical cystectomy.
Sanguedolce 2021 [33]	Retrospective, single-centre; 80, male; prostate cancer (5.6% of 1421 irradiated patients)	3D-CRT 97.5%, 1.8 Gy/fraction; primary 50%, salvage 50%; bladder V70 analysed; mean 43.9 mo (SD 37.5) RT to haemorrhagic cystitis	Conservative management, continuous irrigation, transurethral fulguration, consolidative hyaluronic acid or HBOT, and cystectomy according to severity	Median 36 months	Identify factors associated with hospitalisation and describe treatments and their morbidity	55% managed without admission; 45% hospitalised (64 admissions), with fulguration in 61%, transfusion in 38.9%, and cystectomy in 5, all with complications and 1 death; antiplatelet or anticoagulant therapy (HR 3.30) and higher bladder V70 after salvage RT (HR 1.03 as a continuous variable; cut-off 29%) predicted hospitalisation. Key insight: nearly half of haemorrhagic cystitis presentations required hospitalisation and about one third invasive procedures, with antithrombotic therapy a factor associated with increased risk.
Gatsinga 2024 [34]	Retrospective cohort; 52, male; prostate cancer, RHC needing surgical intervention	Primary RT 78.8%, salvage 21.2%; 36.25–78 Gy (74 Gy in 46%); modality NR; median 64 mo RT to first fulguration	TUBF, HBOT, cystectomy	Median 5.3 years	Evaluate incidence and risk factors for severe RHC	4.5% cumulative incidence; antiplatelet therapy (OR 2.8) and longer interval from RT to first TUBF (OR 1.8) predicted repeat procedures; 5.8% required cystectomy.
Sanguedolce 2022 [35]	Retrospective, single-centre; 51, mixed; pelvic cancers after RT, mainly prostate	Prostate cancer 88.2%; RT primary 31.4%, adjuvant 52.9%, salvage 9.8%, NR 5.9%; median 74 Gy (IQR 68–75); median 31 mo (IQR 21–90) RT to haemorrhagic cystitis	Glycosaminoglycan replacement with hyaluronic acid with or without chondroitin sulphate	Median 59 months	Assess remission of haematuria and predictors of failure	At last follow-up, 70.6% had resolved or only occasional haematuria; 45.1% were readmitted after instillation and 11.8% underwent cystectomy. Key insight: some patients achieve durable control; adjuvant RT and more pre-treatment emergency attendances were associated with failure.
Lin 2017 [36]	Retrospective cohort; 42 (39F/3M); RHC, pelvic RT	Mean 62.6 Gy [29], [30], [32], [33], [37], [38], [39], [40], [41], [42], [43], [44], [45], [46], [47], [48], [49], [50], [51], [52], [53], [54], [55], [56], [57], [58], [59], [60], [61], [62], [63], [64], [65], [66], [67], [68], [69], [70], [71], [72], [73]; modality NR; mean 9.9 y [2], [3], [4], [5], [6], [7], [8], [9], [10], [11], [12], [13], [14], [15], [16], [17], [18], [19], [20], [21], [22], [23], [24], [25], [26] RT to haematuria	HBOT at 2.5 ATA	Mean 20.7 months	Evaluate HBOT clinical outcomes	83.3% complete haematuria resolution; cystoscopic improvement described without statistical testing; no barotrauma or oxygen toxicity.
Dellis 2017 [74]	Prospective cohort; 38, mixed; pelvic cancers, mostly prostate	Mean 63.8 Gy (32–80; NR in 3); modality NR; mean 21.4 mo (1−210) RT to haematuria	Early HBOT as sole primary therapy	Mean 29.3 months	Evaluate response when HBOT is used early in severe haemorrhagic cystitis	Complete response occurred in 86.8% and all complete responders started treatment within 6 months of haematuria onset. Key insight: timing may be critical for maximal benefit.
Gacci 2016 [48]	Prospective pilot study; 30, male; prostate cancer	3D-CRT 66–70 Gy in 33–35 fractions (2 Gy); primary RT 33%, adjuvant RT 67%; symptoms assessed 3 mo after RT	Intravesical hyaluronic acid plus chondroitin sulphate	14 weeks	Evaluate efficacy and safety for post-radiation LUTS and cystitis	Total symptom and bother scores and urgency, frequency, and nocturia subscores improved significantly. Key insight: barrier-restoring therapy may help symptom-predominant bladder injury.
Baboudjian 2022 [49]	Prospective pilot study; 30, mixed; pelvic cancers, mostly prostate	Modality and dose NR; late symptoms >1 y after RT; median 2.3 y (2–2.7) RT to instillation	Weekly intravesical sodium hyaluronate for 6 instillations	1 and 4 weeks after treatment	Assess pain relief and urinary symptom change in late radiation cystitis	Bladder pain improved by 45.8%, with parallel gains in haematuria, frequency, and urgency-related QoL. Key insight: sodium hyaluronate may be useful for pain-predominant disease.
Bracco 2026 [50]	Retrospective cohort; 30, mixed; mixed pelvic cancers, mainly prostate	Modality and dose NR (varied by tumour and site); interval NR	Adelmidrol plus sodium hyaluronate bladder instillations	End of 8-week treatment, with confirmation at 6 months	Assess effect on symptomatic radiation cystitis	Pain, gross haematuria, and urgency improved markedly, while incontinence improved less. Key insight: symptom benefit appears broad, but evidence is retrospective and uncontrolled.
Le Bloa 2023 [51]	Retrospective, single-centre; 29, mixed; radiation-induced haemorrhagic cystitis, mainly after prostate RT	Primary RT 15/27 (EBRT 14, BT 1); post-prostatectomy salvage RT 12/27; dose NR; median 8 y RT to haematuria	GreenLight laser bladder photocoagulation	Median 30 months	Assess control of recurrent haematuria requiring surgery	Absence of recurrent haematuria needing later surgery was achieved in 82.7%. Key insight: bleeding control can be good, but later overactive bladder symptoms were common.
Jaekel 2025 [57]	Retrospective cohort; 28, mixed; radiogenic LUTS after pelvic RT	Modality, dose and interval NR	Intradetrusor botulinum toxin A injection	Not specified	Assess BTX-A effect on radiogenic LUTS and QoL	Significant reduction in micturition frequency, nocturia, and pad use in the whole cohort and by gender, except pad use in men; among dose subgroups, significant reduction in frequency and nocturia only at 200 U; significant ICIQ-LUTSqol improvement across gender and dose subgroups (*n* = 18).
Zhang 2021 [52]	Retrospective, single-centre; 21, female; radiation-induced haemorrhagic cystitis	Modality and dose NR; median 38 mo [8], [9], [10], [11], [12], [13], [14], [15], [16], [17], [18], [19], [20], [21], [22], [23], [24], [25], [26], [27], [28], [29], [30], [31], [32], [33], [34], [35], [36], [37], [38], [39], [40], [41], [42], [43], [44], [45], [46], [47], [48], [74], [75], [76], [77], [78], [79], [80], [81], [82], [83], [84], [85], [86], [87], [88], [89], [90] RT to haematuria	980-nm diode laser coagulation	Median 25 months (range 7–48)	Evaluate efficacy of diode laser coagulation for RHC in women	Complete haematuria resolution in 76.2% after a single session (30-day success rate 95.2%); 19% needed multiple sessions; 1 patient (4.8%) ultimately required radical cystectomy; median haematuria-free interval 16 months.
Bove 2019 [53]	Prospective pilot cohort; 20, mixed; haemorrhagic radiation cystitis after pelvic RT	Mean 60 ± 2.5 Gy; modality NR; RT intent NR; mean 3 ± 1.9 y RT to haemorrhagic cystitis	Endoscopic intravesical fibrin glue	Mean 26.2 months	Assess efficacy and safety of fibrin glue after failure of conventional therapy	Complete response occurred in 80% and no major adverse events were reported. Key insight: fibrin glue is a plausible minimally invasive rescue option in selected patients.
Stamatakos 2022 [37]	Prospective study; 20, mixed; severe radiation haemorrhagic cystitis after pelvic RT	Mean 62.8 Gy [28], [29], [30], [31], [32], [33], [34], [35], [36], [37], [38], [39], [40], [41], [42], [43], [44], [45], [46], [47], [48], [49], [50], [51], [52], [53], [54], [55], [56], [57], [58], [59], [60], [61], [62], [63], [74], [79], [80], [81], [82], [83], [84], [85], [86], [87], [88], [89], [90]; modality NR; mean 18.1 mo (1–210) RT to haematuria	HBOT as primary treatment	Mean 24.6 months	Evaluate response and safety in high-grade haemorrhagic cystitis	Complete response occurred in 85% and partial response in 15%, with no treatment complications. Key insight: earlier treatment after haematuria onset appeared linked to better response.
Chong 2016 [47]	Retrospective review; 12, mixed; RHC, urological cancers	Modality, dose and interval NR	HBOT	Mean 443 days	Assess HBOT efficacy in RHC	Complete resolution in 50%, partial in 17% (67% overall improvement).
Ziegelmann 2017 [54]	Retrospective series; 8, mixed; radiation haemorrhagic cystitis after prostate or cervical RT	EBRT 100%; dose NR; median 98 mo (IQR 71–126) RT to formalin instillation	Intravesical formalin, 1% to 4%	Median 8 months	Evaluate inpatient haematuria control with formalin	Six of eight patients achieved haematuria resolution during the same admission, and 3 underwent cystectomy (1 non-responder during the same admission; 2 responders later); the other non-responder was managed with nephrostomy tubes. Key insight: formalin can control severe bleeding quickly, but later bladder dysfunction and cystectomy remained concerns.
Abbruzzese 2023 [55]	Prospective pilot cohort; 9, male; prostate cancer	Prostate RT; modality and dose NR; median 10 mo [6], [7], [8], [9], [10], [11], [12], [13], [14], [15], [16], [17], [18], [19], [20], [21], [22], [23], [24], [25], [26], [27], [75], [76], [77], [78], [79], [80], [81] RT to haematuria	Intravesical allogeneic platelet lysate, 24 instillations over 3 months, after failed clot evacuation and electrocoagulation	Median 56 months	Assess resolution of haematuria and regeneration of bladder mucosa	All 9 patients had complete resolution of haematuria at 3 months (residual low-intensity urinary symptoms in up to 3 resolved subsequently), with cystoscopic and histological normalisation of the mucosa; no recurrence at a median of 56 months and cystectomy avoided in 4 candidates. Key insight: regenerative instillation may offer an alternative where HBOT is unavailable, but evidence is limited to one small series.
Kam 2026 [56]	Prospective pilot case series; 15, male; prostate cancer	Prostate RT: primary 47%, adjuvant or salvage 53%; modality and dose NR; median 7 mo (1.5–17) RT to treatment	Cystoscopic application of RADA16 self-assembling peptide under CO2 insufflation after failed standard treatment	Median 4 months	Describe technique, safety, and efficacy for refractory haematuria	Haematuria improved in 93% at 6 weeks; Clavien Grade ≥ 3 complications in 27% within 90 days, including repeat application or diathermy and 1 salvage cystectomy. Key insight: a promising haemostatic and regenerative option, but follow-up is too short to judge durability.

Abbreviations: ATA, atmospheres absolute; BTX-A, botulinum toxin A; ED, emergency department; EPIC, Expanded Prostate Cancer Index Composite; HBOT, hyperbaric oxygen therapy; HR, hazard ratio; ICIQ-LUTSqol, International Consultation on Incontinence Questionnaire Lower Urinary Tract Symptoms quality of life module; LUTS, lower urinary tract symptoms; OR, odds ratio; QoL, quality of life; RHC, radiation haemorrhagic cystitis; RIC, radiation-induced cystitis; RICH-ART, Radiation-Induced Cystitis treated with Hyperbaric oxygen therapy trial; RT, radiation therapy; TUBF, transurethral bladder fulguration. NR, not reported; 3D-CRT, three-dimensional conformal radiation therapy; IMRT, intensity-modulated radiation therapy; SBRT, stereotactic body radiation therapy; HDR, high dose rate; LDR, low dose rate; fr, fractions; mo, months; y, years.

**Table 4:** Characteristics of studies of reconstructive management for late urinary side effects after pelvic RT.

Author, year	Study design; n, sex; disease site	RT characteristics	Intervention & management strategy	Follow-up	Primary objective	Main findings & key insight
Angulo 2023 [41]	Retrospective multicentre cohort (11 centres); 223, male; ATOMS after prostatectomy + RT	Prostatectomy + adjuvant RT; salvage prostatectomy after RT 5.4%; prior BT 0.9%; dose NR; interval NR	Silicone-covered scrotal port ATOMS implant	Median 30 months	Evaluate ATOMS efficacy/safety after combined RP + RT	42.6% achieved dryness; 84% of the 125 with data self-reported satisfied; baseline incontinence severity the dominant predictor (65.6% dry if mild vs 24% if severe).
Voelzke 2021 [19]	Retrospective, multi-institutional; 137, male; prostate cancer	BT 43.8%, EBRT 38.0%, EBRT+BT 9.5%, other single-modality RT 8.7%; adjuvant or salvage RT after prostatectomy 14.6%; dose NR; interval NR; >1 modality associated with recurrence on univariable analysis only	EPA urethroplasty for RT-associated bulbomembranous stenosis	Mean 32.3 months	Assess unobstructed instrumentation-free voiding after EPA urethroplasty	Success was 86.9%; 32.1% developed stress incontinence and 21.9% later required AUS. 16.1% experienced post-op complications. Combined modality RT was associated with stricture recurrence on univariable (*p* = 0.004) but not multivariable analysis; stenosis length and age were independent predictors. Key insight: EPA offers the strongest patency signal, but functional cost remains important.
Queissert 2023 [40]	Retrospective comparative cohort; 123 (61 RT-only/62 RT + dBO), male; AMS 800, RT ± devastated bladder outlet	Primary RT 8.2%, adjuvant or salvage 91.8% (73 with data); BT 1.6–3.2%; dose NR; 74–86 mo RT to AUS	AMS 800 implantation, comparative cohort	Median 21 months	Compare AUS outcomes in RT-only vs RT + dBO patients	dBO group: more revision (43.1% vs 26.3%, *p* = 0.05), mainly erosion; ≥2 prior desobstructions independently predicted revision (HR 2.8).
Rotz 2025 [88]	Retrospective cohort, time-since-RT analysis; 99, male; first-time AUS, prior pelvic radiation	EBRT + prostatectomy 89/99, BT + TURP 6, EBRT alone 1; RT + other 3; 66 Gy/33 fr in nearly all with data (52/99); median 90 mo (IQR 41–129) RT to AUS	AUS implantation, time-since-RT analysis	Median 31 months	Determine if time since radiation predicts AUS complications	Time since RT not associated with device failure (HR 1.00, *p* = 0.63); larger cuff size protective (HR 0.27); higher ASA class increases risk (HR 5.28); 5-year device survival 87%.
Rozanski 2022 [59]	Retrospective, two-institution; 91, male; predominantly prostate cancer (97%)	EBRT 63%, BT 26%, EBRT+BT 5%, proton 5%; primary RT 81%, adjuvant or salvage 19%; dose NR; interval NR; failure highest after EBRT+BT (60%)	Conservative management with dilation, DVIU, and or intermittent catheterisation	Median 15 months	Assess whether conservative treatment can avoid reconstruction or permanent catheter	Eighty percent avoided reconstruction or permanent catheter. Key insight: non-definitive management can be reasonable in frail, comorbid patients, though UTIs and retention remain common.
Malshy 2024 [66]^c^	Retrospective comparative cohort; 85 (33 TC/52 SP), male; virgin AUS, prior pelvic RT	RT alone or prostatectomy with adjuvant or salvage RT; modality and dose NR; median 86.5 vs 39.4 mo RT to AUS (transcorporal vs standard)	Transcorporal (TC) vs standard placement (SP) cuff	51.9 vs 80.4 months	Compare device lifespan by placement technique	Device removal 36.4% (TC) vs 55.8% (SP); not significant (HR 0.717, *p* = 0.35); survival at 10 yr: 62.1% vs 46.7%.
Policastro 2021 [58]^b^	Retrospective, multi-institutional; 79, male; prostate cancer	EBRT 45.6%, BT 16.5%, EBRT+BT 12.7%, prostatectomy+EBRT 25.3%; dose NR; median 48 mo (IQR 14–108) RT to stricture	Dorsal onlay buccal mucosal graft urethroplasty	Median 21 months	Assess recurrence and de novo stress incontinence and patient related outcomes	Recurrence occurred in 17.7%; with a median IPSS improved from 21 to 6 and de novo stress incontinence occurred in 8.1%. Key insight: dorsal grafting may offer favourable symptom benefit in selected patients.
Fuchs 2017 [61]	Retrospective review; 72, male; prostate cancer(*n* = 71), colorectal cancer (*n* = 1)	EBRT 45.8%, BT 36.1%, EBRT+BT 12.5%, proton 4.2%; dose NR; median 6.0 y (IQR 3–9) RT to repair	EPA urethroplasty	Median 33.5 months	Assess recurrence after EPA for recurrent radiation strictures	Recurrence occurred in 23.6% overall. Key insight: later-era success was higher (86% vs 70%, *p* = 0.07), but stress incontinence remained common (34.7%).
Malshy 2024 [67]^c^	Retrospective, bi-institutional; 72, male; AUS after pelvic RT, prostate cancer	EBRT 76.4%, BT 6.9%, EBRT+BT 13.9%, NR 2.8%; dose NR; AUS timing analysed at 2–5 y after RT	Timing of first AUS placement relative to RT	Median 57.5 months	Determine optimal timing window for AUS placement post-RT	50% device Rev./Exp; no significant difference by 2/3/4/5-year timing cutoffs, but consistent trend toward higher erosion/infection the later the insertion.
Toia 2019 [18]^d^	Retrospective cohort; 54, mixed; pelvic malignancies, mainly cervical and colorectal cancers	Modality identified in 26/54, of whom 69% had EBRT+BT boost; dose NR; mean 13 y RT to reconstruction	Mixed reconstructive surgery for fistula, ureteric repair, bladder reconstruction, or diversion	Median 22 months	Describe reconstructive needs and functional outcomes after late pelvic RT injury	Fistula closure was difficult, bowel interposition was often needed for ureteric repair, and GFR stabilised or improved in 87% but re-intervention rate of 28% and significant morbidity in 17%. Key insight: radiation reconstruction is heterogeneous, and reintervention is common.
Carilli 2024 [77]	Retrospective, multi-institutional; 53, mixed; pelvic malignancies, most commonly cervical cancer	EBRT 100%, median 48 Gy; concomitant chemotherapy recorded (independent predictor of recurrence); median 6 mo (IQR 4–10) RT to symptom onset	Endourologic treatment or robotic ureteral reconstruction by stricture complexity	Median 12 months	Compare outcomes by reconstructive approach	Endourology achieved 59% overall success versus 96% for robotic reconstruction; recurrence was 15.1% and eGFR improved. Stricture length over 2 cm (OR 6.4) and concomitant chemotherapy at the time of radiation therapy (OR 8.9) were univariable predictors of recurrence; chemotherapy remained an independent predictor on multivariable analysis (OR 7.8). Key insight: stricture length (endourology for short and mid-proximal stricture, robotic-assisted others) and complexity should drive treatment choice.
Miller 2022 [87]	Retrospective comparative cohort; 45, male; prostate cancer, all with prior pelvic irradiation	Salvage RT 74–89%, adjuvant 11–19%; median 69.8 vs 67 Gy (standard vs transcorporal); median 62 vs 108 mo RT to AUS	Initial AUS placement, transcorporal (*n* = 27) versus standard (*n* = 18) approach	Median 32 months (transcorporal) and 39 months (standard)	Compare major complications and functional outcomes between transcorporal and standard AUS placement in irradiated men	Major complications occurred in 14.8% with transcorporal versus 56% with standard placement (*p* = 0.01); explant rate 7.4% versus 39% (*p* = 0.02); revision rate 7.4% versus 33% (*p* = 0.04). Postoperative IPSS and QoL scores also favoured transcorporal placement (*p* = 0.001 and p = 0.01). Key insight: transcorporal placement may reduce major complications when AUS is used after pelvic radiation, but selection was not randomised.
Sterling 2024 [86]^b^	Retrospective, multi-institutional; 45, male; prostate cancer	Prostatectomy + RT in all (primary prostatectomy 89%, salvage 11%); median 73 Gy (IQR 69–76) in 26/45; median 48 mo (IQR 28.5–88.5) RT to symptoms	Dorsal onlay buccal mucosal graft urethroplasty	Median 21 months	Assess recurrence, continence outcomes and patient-related outcomes	Seven recurrences occurred and no de novo incontinence was observed. Patients reported improvements in symptoms and QoL. Key insight: dorsal onlay grafting may give lower de novo incontinence than excisional urethroplasty (no head-to-head data), but longer follow-up is needed.
Ceballos 2025 [64]^a^	Retrospective, multi-institutional; 37 analysable, male; pelvic RT, primary site NR	Modality, dose and interval NR	Optilume drug-coated balloon dilation for posterior urethral stenosis	Mean 179 days	Assess early efficacy and safety of Optilume	Freedom from repeat intervention was 81.1% (30/37); complications in 10.7% (6/56 treated), none exceeding Clavien IIIb. Key insight: early results are promising, but follow-up is short.
Thomas 2026 [71]	Retrospective cohort; 36, mixed; mixed pelvic malignancies	Group 2: primary RT 35%, postoperative or salvage RT 65%; modality and dose NR; median 4 mo (group 1) and 66 mo (group 2) RT to augmentation	Augmentation enterocystoplasty, often with ureteric reimplantation	Median 13 years for extirpative group and 5 years for delayed group	Describe short and long-term outcomes after augmentation	Outcomes were best when augmentation was done at extirpative surgery, while delayed augmentation had catheter dependence, fistula, and later cystectomy. Key insight: bladder preservation is possible, but long-term functional uncertainty is substantial.
Rourke 2016 [62]	Retrospective cohort; 35, male; prostate cancer	EBRT 57% (20/35), BT 43%; post-prostatectomy RT excluded; dose NR; mean 4.9 y [1], [2], [3], [4], [5], [6], [7], [8], [9], [10], [11], [12], [13], [14], [15], [16] RT to stenosis diagnosis	Urethroplasty with EPA or tissue transfer after failed endoscopic treatment	Mean 50.5 months	Assess urethral patency after reconstruction	Overall success was 85.7%. Key insight: reconstruction is effective, but adverse change in continence (25.7%) and erectile function (30.4%) and persisting storage symptoms (40.0%) were common.
Huang 2026 [79]	Retrospective, multi-institutional; 35, female; pelvic malignancies, mainly cervical cancer (85.7%)	Cervical cancer 85.7%; modality, dose and interval NR	Robotic ileal ureter replacement with or without augmentation ileocystoplasty	Median 20 months	Assess feasibility and efficacy of robotic ileal ureter replacement	Clinical success was 97.1%, 82.9% had bilateral disease, and eGFR improved with no Clavien Grade ≥ 3 complications. Key insight: even long bilateral disease can be reconstructed in expert hands.
Asghar 2021 [69]	Retrospective cohort; 32 patients and 35 ureteral units, mixed; endometrial, cervical, colorectal, and prostate cancers	Modality, dose and interval NR	Robotic ureteral reconstruction with tailored techniques	Median 13 months	Assess feasibility and effectiveness of robotic reconstruction	Clinical and radiologic success was 88.2% (30 of 34 evaluable ureteral units; one perioperative death excluded). Key insight: robotic reconstruction is feasible in experienced centers despite irradiated tissue.
Masopeh 2025 [65]^a^	Retrospective; 30, male; radiation-related urethral stenosis after pelvic RT	Modality, dose and interval NR	Optilume drug-coated balloon dilation	Median 353 days	Assess short-term success of drug-coated balloon treatment	Success was 80%, but 20% of previously continent men developed new stress incontinence. Key insight: less invasive treatment may trade durability for continence risk.
Xu 2024 [78]	Retrospective single-arm cohort; 27 (33 stents), female; ureteral stricture, gynaecologic RT ± chemo	Gynaecologic RT in 25 fractions (all), afterloading BT in 10/27; chemotherapy in 88.9%; total dose NR; mean 5.35 ± 4.14 y RT to stent	Thermo-expandable metallic stent (Memokath)	Patency data to 29 months	Assess safety/efficacy of metallic stents for post-RT ureteral stricture	Patency 92.4% at 8mo, 77.4% at 12mo, 67.7% at 29mo; complications in 6/27 (22.2%), 5 of them late (refractory UTI, migration, stent intolerance) and requiring stent removal; hydronephrosis (*p* < 0.001) and GFR (*p* = 0.016) significantly improved, creatinine did not (*p* = 0.059).
Guan 2025 [68]	Retrospective case series; 26, female; cervical (73.1%) and endometrial (26.9%) cancer	Pelvic RT, setting and modality NR; median 37.5 Gy (IQR 30–46.25); median 20 fractions (IQR 15.75–25.25); interval NR	Trans-retropubic mid-urethral sling with deliberately non-tension-free, individualised tape tension	1 year	Assess continence outcomes and complications after mid-urethral sling for stress urinary incontinence following pelvic RT	80.8% achieved treatment success at 1 year; ICIQ-SF sum score improved from median 20.5 to 4.5 (*p* < 0.01 at 2 weeks, 6 months, and 1 year). No sling exposure at 1 year; 19.2% had transient postoperative dysuria, one requiring sling release. Key insight: this is the only included study addressing female stress urinary incontinence after pelvic RT, and suggests deliberate sling tension, rather than the tension-free principle used in non-irradiated patients, may be important for success.
Gani 2020 [42]	Prospective pilot study; 23, male; prostate cancer with severe stress urinary incontinence	Prostatectomy + adjuvant or salvage RT 65.2%; primary RT 17.4%; HDR BT 8.7%; RT + salvage prostatectomy 8.7%; dose NR; median 2 y (IQR 1–3.5) last treatment to AUS	AUS implantation with autologous rectus fascial wrap around the urethra	Median 32 months	Assess erosion, complications, and continence after AUS with fascial wrap	Erosion occurred in 4.3% (1/23); of the 22 with a device in situ, 77.3% achieved complete continence and 22.7% social continence. Key insight: continence reconstruction is feasible in irradiated men, but prosthetic risk remains central.
Barnard 2023 [63]	Retrospective cohort; 23, male; prostate cancer	Modality and dose NR; median 58.8 mo (1–165) RT to obstructive symptoms	Anastomotic urethroplasty	Mean 73.1 months	Assess long-term anatomic and patient-reported outcomes	Long-term success was 91.3%, and satisfaction was 91.3% despite sexual side effects, and 95.7% of patients stated they would choose surgery again. Key insight: durable control is possible despite continence and sexual side effects.
Li 2021 [76]	Retrospective cohort; 18, female; cervical (*n* = 15) and endometrial (*n* = 3) cancer	EBRT 45–50 Gy (*n* = 4) or BT, HR-CTV D90 85–90 Gy (*n* = 8), otherwise NR; median 12 mo (2–106) RT to detection of stricture	Bilateral ileal ureter substitution using reverse 7 configuration	Median 24.1 months	Assess success of bilateral ileal ureter substitution	Success was 94.4%; 8 minor complications occurred in 5 patients (28%) and major complications in 2 (11%). Key insight: Ileal substitution is successful for bilateral radiation strictures in selected female gynaecologic cohorts and strict post-operative management is essential
Neulander 2019 [70]	Retrospective case series; 11, mixed; cervical, rectal, rectosigmoid, and anal cancers	RT with or without radical hysterectomy 7 (BT in 1), preoperative RT for rectal cancer 3, anal cancer 1; modality otherwise NR; dose NR; interval NR	Ureterolysis, ureterotomy, and omental sleeve wrap, with one Boari flap reimplantation	Median 56 months	Assess patency after surgery for ureteral obstruction due to radiation-related pelvic fibrosis	Ureteral patency was restored in 9 of 11 patients and 18 of 20 renal units. Key insight: selected ischemic upper-tract obstruction can sometimes be managed without bowel substitution.
Smith and Han 2020 [60]	Retrospective, single-surgeon; 16, male; prostate cancer	BT 37.5%, EBRT 12.5%, SBRT 18.75%, BT + EBRT 18.75%, BT + cryotherapy 6.25%, BT + SBRT 6.25%; dose NR; interval NR	Endoscopic management (TURP, incision, dilation) or urethroplasty for post-radiation prostatic urethral stenosis	Mean 2.6 years	Report outcomes and complications after intervention	TURP required further intervention in 48%, dilation in 90%, and urethrotomy or laser vaporisation in all cases; neither of 2 urethroplasties recurred. Incontinence in 10 of 16, with 5 AUS placements, 3 chronic catheterisations, and 1 fistula requiring diversion. Key insight: endoscopic management can stabilise prostatic stenosis, at a high cost in incontinence and repeat intervention.
Polyakov 2016 [80]	Retrospective cohort; 192, female; gynaecologic cancers, predominantly cervical	Modality, dose and interval NR	Extravesical ureteroneocystostomy for defects <4 cm (*n* = 49), or Boari flap with (*n* = 45) or without (*n* = 98) antireflux protection for defects of 4–15 cm	Median 38.2 months	Evaluate effectiveness of reconstruction of distal ureteral strictures	Patency restored in 96%, 95.5%, and 100% respectively, with recurrences salvaged by reoperation; renal function stable in all groups; Boari flap without antireflux protection carried 5.1% clinically significant reflux and 18.4% pyelonephritis versus 6.7% with antireflux protection. Key insight: the largest series of radiation-related distal ureteral strictures shows reimplantation is highly effective; pyelonephritis was numerically, but not significantly, lower with antireflux protection, and within the Boari flap groups the only recurrences (4.4%) occurred with antireflux protection.
Huang 2024 [81]	Retrospective cohort; 7, female; cervical (*n* = 5), endometrial, and colorectal cancer	Postoperative RT (all); modality and dose NR; interval NR	Bilateral ileal ureter replacement with ileocystoplasty (open, laparoscopic, or robot-assisted)	3–28 months	Assess feasibility and mid-term outcomes for bilateral long-segment strictures with bladder contracture	No Clavien Grade ≥ 3 complications; creatinine stable (88 to 95 μmol/L) with no renal deterioration; bladder capacity increased from 99 to 249 mL; mean hospital stay 27 days. Key insight: combined ileal reconstruction is feasible for concurrent ureteral and bladder injury, but the series is small with short follow-up.

Abbreviations: AMS, American Medical Systems; ASA, American Society of Anesthesiologists; ATOMS, adjustable trans-obturator male system; AUS, artificial urinary sphincter; dBO, devastated bladder outlet; DVIU, direct vision internal urethrotomy; EPA, excision and primary anastomosis; GFR, glomerular filtration rate; HR, hazard ratio; ICIQ, International Consultation on Incontinence Questionnaire; ICIQ-SF, International Consultation on Incontinence Questionnaire Short Form; IPSS, International Prostate Symptom Score; PROMs, patient-reported outcome measures; QoL, quality of life; RP, radical prostatectomy; RT, radiation therapy; SP, standard placement; TC, transcorporal; UTI, urinary tract infection. NR, not reported; 3D-CRT, three-dimensional conformal radiation therapy; IMRT, intensity-modulated radiation therapy; SBRT, stereotactic body radiation therapy; HDR, high dose rate; LDR, low dose rate; fr, fractions; mo, months; y, years.

^a,b,c,d,^ Studies sharing a superscript letter report overlapping patient populations. ᵈ Studies sharing this superscript report overlapping patient populations with a study in Table 5; See Appendix C

**Table 5:** Characteristics of studies of salvage and end-stage management for late urinary side effects after pelvic RT.

Author, year	Study design; n, sex; disease site	RT characteristics	Intervention & management strategy	Follow-up	Primary objective	Main findings & key insight
Bassett 2017 [38]	Retrospective, multi-institutional; 100, male; prostate cancer	Combined-modality 81% (prostatectomy+EBRT 49%, BT + EBRT 17%, HDR BT + boost 12%, other 2%); single-modality 19% (EBRT 12%, LDR BT 4%, HDR BT 2%, proton 1%); dose NR; median 8 y (IQR 5–12) RT to diversion	Urinary diversion, usually conduit and often with cystectomy	Median 16.3 months	Describe perioperative and longer-term morbidity after diversion	Clavien Grade ≥ 3a short-term complications occurred in 35%, with later surgery needed in 22% for long term complications. Key insight: diversion is effective but carries major short and long term burden.
Myers 2024 [39]	Retrospective cohort; 69, male; prostate cancer	Adjuvant or salvage RT after prostatectomy 34.8%; EBRT+BT 23.2%, EBRT 20.3%, HDR BT 14.5%, LDR BT 2.9%, proton 1.4%; salvage RT or cryotherapy after RT 8.7%; dose NR; median 7.3 y (IQR 3.2–12.5) RT to diversion	Urinary diversion tailored to anatomy and severity for fistula or localised radiation injury	Median 665 days	Compare diversion outcomes by fistula status	Fistula patients had longer operations, more abdominal or perineal approaches, and higher Clavien Grade ≥ 3 complications than non-fistula patients. Key insight: fistula status marks a higher-risk salvage population.
Woodle 2026 [84]	Prospective cohort; 55 enrolled and 47 paired surveys, mixed; pelvic radiation-related urologic injury, predominantly after prostate RT (82%)	Modality, dose and interval NR	Urinary diversion using conduit or continent options	Median 47.7 months	Assess long-term HRQoL after diversion and decision regret	PROMIS physical and mental health scores improved by 7.0 and 7.1 points respectively, exceeding minimally clinically important differences. GUPI total score fell significantly from 21 preoperatively to 7 at early and 5 at late follow-up, and decision regret remained low at 12.6 early and 10.4 late. Key insight: prospective QoL evidence supports diversion in severe disease.
Tachibana 2021 [85]	Retrospective cohort; 45, mixed; pelvic cancers with refractory radiation cystitis	EBRT 53%, BT 7%, EBRT+BT 18%, proton 4%; modality NR in 18%, dose NR; mean 10.5 y RT to diversion	Urinary diversion with or without cystectomy	Mean 14.6 months	Assess effect of surgery on procedures, admissions, transfusions, and visits	Procedures fell from 3.0 to 0.2, hospitalisations from 2.3 to 0.6 (among 21 hospitalised beforehand), and transfusions from 7.8 to 0.8 units. Key insight: diversion can markedly reduce treatment burden even when morbidity is substantial.
Holm 2025 [73]	Retrospective observational study; 34, male; predominantly prostate cancer	Prostatectomy + adjuvant or salvage EBRT 44.1%, EBRT 11.8%, EBRT + BT, cryotherapy or HIFU 8.8%, TURP + adjuvant EBRT 5.9%; dose NR; median 140 mo (26–312) primary treatment to diversion	Salvage urinary diversion after failed AUS and devastated bladder outlet	Median 18 months	Describe characteristics and outcomes of diversion as last resort after failed outlet reconstruction	Post-diversion complications occurred in 32%, and 81.3% of respondents said they would undergo diversion again. Key insight: diversion can remain acceptable even after repeated prior reconstructive failure.
Smith 2020 [46]	Retrospective cohort; 34, female; gynaecologic malignancies after RT, mainly cervical cancer (52.9%)	EBRT 100%: surgery+EBRT+chemotherapy 35.2%, surgery+EBRT 32.4%, EBRT+chemotherapy 32.4%; dose NR; median 9.5 y (IQR 4–20) RT to diversion	Urinary diversion with or without cystectomy	Median 13 months	Identify predictors of high-grade complications after diversion	Ninety-day complications occurred in 79.4%, high-grade complications in 26.5%, and readmission within 30 days = 32.4%. Key insight: diversion for gynaecologic radiation injury carries high early morbidity.
Piazza 2021 [90]	Retrospective cohort; 32, male; prostate cancer	Salvage RT 44%, primary 28%, adjuvant 28%; modality NR; median 70 Gy (IQR 65–76); median 11 y (IQR 6–15) RT to cystectomy	Robot-assisted cystectomy with intracorporeal urinary diversion	Median 30 months	Assess perioperative and late outcomes after robotic salvage cystectomy	Major early complications occurred in 28% and late complications in 46%, with no perioperative mortality. Key insight: robotic salvage surgery remains morbid despite technical advantages.
Al Hussein Al Awamlh 2015 [75]	Retrospective cohort with health-related quality of life survey; 29, mixed; refractory benign pelvic radiation disease, mostly after prostate RT (75.9%)	EBRT 69%, BT 6.9%, EBRT+BT 24.1%; dose NR; median 87 mo (IQR 39–150) RT to refractory symptoms	Cystectomy with urinary diversion	Median 37.3 months	Assess HRQoL after cystectomy	Significant improvements based on Medical Outcomes Study Short Form version 2 in pain, social function, general health, and cumulative mental health. SF-36 pain improved from 57.1 to 85.8 and cumulative mental health from 52.7 to 75.1, despite 30-day complications in 65.5% (Clavien Grade III in 20.7%). Key insight: salvage surgery can improve HRQoL, not only anatomical control.
Andrews 2021 [45]	Retrospective case series; 25, male; prostate cancer	Prostatectomy 15 (adjuvant EBRT 5, salvage EBRT 10), EBRT 7, BT 2, EBRT+BT 1; salvage cryotherapy after EBRT 3, salvage BT after EBRT 1; dose NR; median 11 y (IQR 6–16.5) treatment to fistula	Pubectomy with urinary reconstruction, usually cystectomy with conduit diversion; flap interposition in 52%	3 months	Describe outcomes after surgery for urosymphyseal fistula	Pain improved in 96% and resolved in 92%, with ECOG improvement despite substantial short-term morbidity. Key insight: symptom relief can be dramatic even when perioperative burden is high.
Brändstedt 2023 [89]	Retrospective case series; 24, male; predominantly prostate cancer, one bladder cancer	Primary EBRT (70 Gy/35 fr or 78 Gy/39 fr; pelvic nodes in 17%), LDR BT 4%, salvage EBRT after prostatectomy 17%, salvage cryotherapy after EBRT 8%; median 5 y (IQR 2–7) RT to fistula diagnosis	Diversion with or without cystectomy or pelvic exenteration, plus antibiotics	Median 22 months	Describe presentation and outcomes in urosymphyseal fistula after pelvic RT	Recurrent osteomyelitis was more common when cystectomy was not performed, and conservative catheter-based care carried high infection mortality. Key insight: delayed or incomplete salvage may leave ongoing sepsis risk.
Toia 2020 [82]^d^	Retrospective cohort; 20, female; cervical, ovarian, urethral, endometrial, and rectal cancers	EBRT 100%, BT boost 30%; dose NR; mean 19 y (0–40) RT to fistula	Surgical vesicovaginal fistula repair or urinary diversion tailored to anatomy	Not explicitly reported	Assess closure rates and functional outcomes in radiation-associated vesicovaginal fistula	Final closure was only 25% and 70% required diversion. Key insight: spontaneous radiation fistula is rarely amenable to durable repair.
Walach 2024 [20]	Retrospective case series with PROM follow-up; 20, mixed; mainly prostate cancer (85%), with some colorectal, anal, and cervical cancers	EBRT 95%, BT 5%; median 75 Gy (IQR 73–99) over 35 d; median 4.0 y (IQR 0.9–6.5) RT to diagnosis	Extirpative salvage surgery with cystectomy and ileal conduit plus bone resection or debridement	Mean 23 months for PROM follow-up after cystectomy	Describe diagnostic features and postoperative patient-reported outcomes	Imaging changes evolved over time and all patients underwent extirpative surgery; QoL after surgery was variable. Key insight: earlier recognition may help before disease becomes advanced.
Inouye 2022 [83]	Retrospective case series; 18, male; prostate cancer	Prostatectomy + adjuvant or salvage EBRT 55.6%, EBRT+BT 33.3%, EBRT + salvage cryotherapy 11.1%; dose NR; median 16 y (IQR 10.5–19) RT to osteomyelitis	Cystectomy, urinary diversion, and pubic symphysis resection	Median 15 months	Assess change in physical and mental health by Short Form-12	SF-12 physical score improved significantly from 30.6 to 45.5, and morphine equivalents decreased significantly from 119.4 to 30.5. Key insight: QoL gains can be measured directly after salvage surgery.
Pereira 2025 [72]	Retrospective cohort; 17, mixed; gynaecologic, prostate, and rectal cancers with refractory radiation haemorrhagic cystitis	RT for gynaecologic (64.7%), prostate (EBRT, 23.5%) and rectal (11.8%) cancer; modality otherwise NR; dose NR; median 65 mo (IQR 35–182) RT to cystectomy	Supratrigonal cystectomy with bladder mucosa fulguration and urinary diversion	Median 28 months	Report outcomes of supratrigonal cystectomy as last-line treatment	Major complications occurred in 29.4%, with 1- and 3-year survival of 93.3% and 83.3%. Key insight: this is a true last-line option for otherwise unmanageable haemorrhage.
Bugeja 2016 [44]	Retrospective case series; 16, male; prostate cancer	Primary RT 8/16, post-prostatectomy RT 8/16; combinations of BT, EBRT and cryotherapy 31.3%; salvage cryotherapy 3; dose NR; mean 94.2 mo (12–216) initial cancer treatment to presentation	Excision of fistula and involved pubic bone with omentoplasty, then reconstruction or cystectomy as needed	Early and 30-day outcomes emphasized	Describe presentation and results of surgery for pubic symphysis fistulation	Thirteen of fifteen surgically treated patients were cured of symptoms, while conservative treatment rarely worked. Key insight: this is a surgical disease in most patients.
Lavien 2017 [43]	Retrospective case series; 16, male; prostate cancer	EBRT+BT 37.5%, EBRT 18.8%, prostatectomy + adjuvant EBRT 18.8%, prostatectomy + salvage EBRT 12.5%, EBRT + salvage cryotherapy 12.5%; dose NR; median 8 y (IQR 1.8–14) prostate cancer treatment to osteomyelitis diagnosis	Pubic bone resection with extirpative urinary surgery or diversion as needed	Median 9.4 months	Assess change in pain intensity after surgery	Pain fell from median 5.5 to 0 and narcotic use declined significantly. Key insight: surgery offers durable pain relief where conservative care does not.
Keogh 2023 [91]	Retrospective case series; 13 (7F/6M); end-stage bladder, radiation damage, multiple pelvic malignancies	Modality and dose NR; 0.5–37 y RT to diversion	Urinary diversion (ileal conduit 6, continent neobladder 6, ureterostomy 1)	Mean 27 months	Evaluate diversion outcomes and patient satisfaction for failed radiated bladder	High satisfaction; pain/UTI improved in 7 of 10 questionnaire respondents; 8 of 10 would choose surgery again and elect to have it sooner.

Abbreviations: AUS, artificial urinary sphincter; ECOG, Eastern Cooperative Oncology Group; GUPI, Genitourinary Pain Index; HRQoL, health-related quality of life; PROM, patient-reported outcome measure; PROMIS, Patient-Reported Outcomes Measurement Information System; QoL, quality of life; RT, radiation therapy; SF-36, Medical Outcomes Study 36-Item Short Form Health Survey; UTI, urinary tract infection. NR, not reported; 3D-CRT, three-dimensional conformal radiation therapy; IMRT, intensity-modulated radiation therapy; SBRT, stereotactic body radiation therapy; HDR, high dose rate; LDR, low dose rate; fr, fractions; mo, months; y, years.

ᵈ Studies sharing this superscript report overlapping patient populations with a study in Table 4; See Appendix C

## Results

Fig. 1 summarises the study selection process, which resulted in 70 included publications. The RICH-ART hyperbaric oxygen trial contributed two publications, the primary report [25] and its five-year follow-up [26], which describe the same patient population. These were charted as one study, so the 70 publications represent 69 distinct studies. Four further pairs of publications reported partially overlapping, but not identical, patient populations. Each pair shared some patients while differing in recruitment period, eligibility criteria or cohort composition. These pairs were therefore retained as separate analyses and are identified in Table 3, Table 4, Table 5 and Appendix C. Of the 69 studies of previously irradiated patients, 24 addressed bladder-preserving, 28 reconstructive, and 17 salvage or end-stage management.

**Fig. 1:**
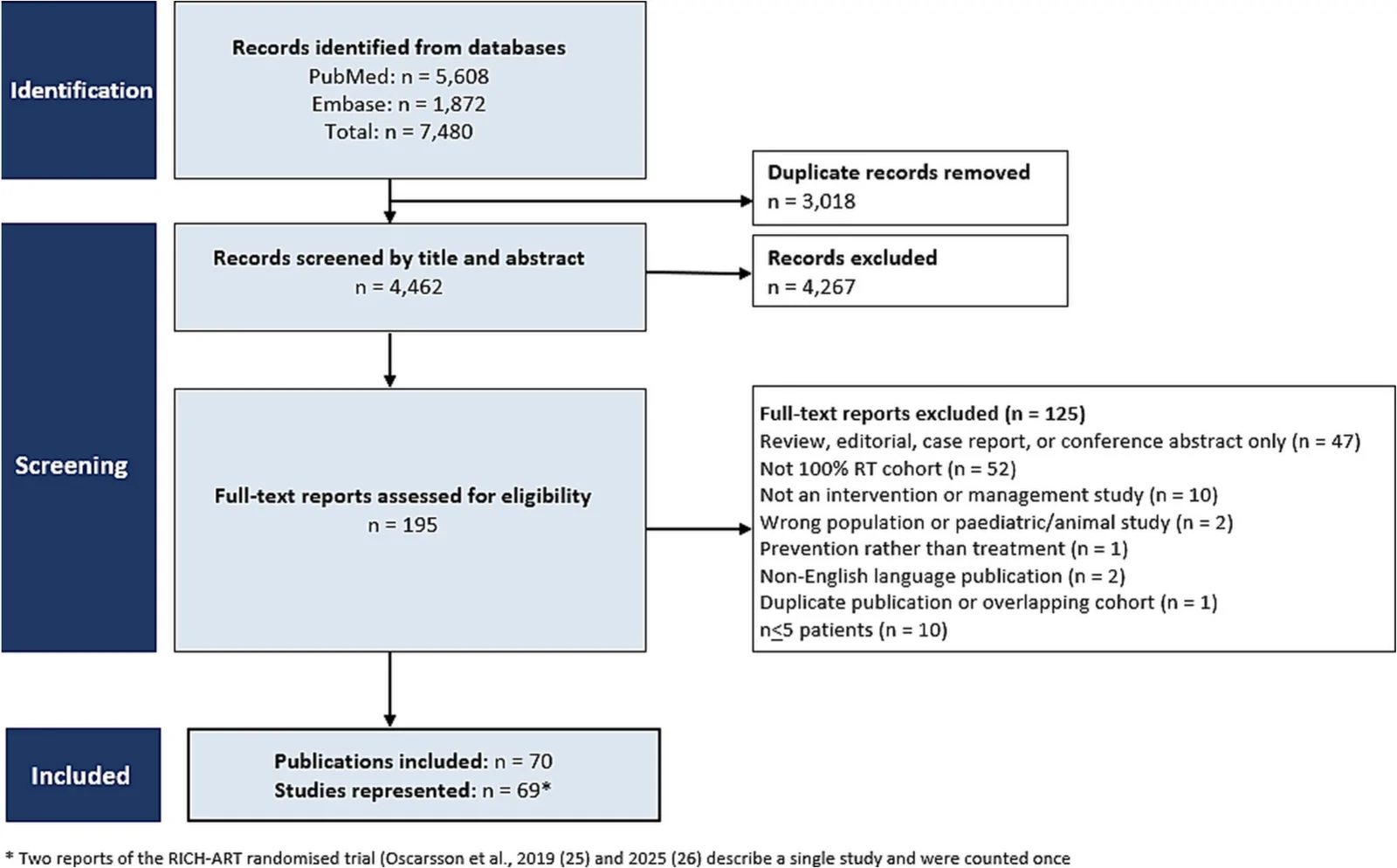
PRISMA-ScR flow diagram.

Under RoB 2, the hyperbaric oxygen trial was judged at high risk of bias, arising from a patient-reported primary outcome in an unblinded design [25], [26]; the sodium hyaluronate trial raised some concerns [27] (Appendix B).

### Characteristics of sources of evidence

Of the 69 studies (70 publications), only 2 were randomised controlled trials, a trial investigating hyperbaric oxygen therapy (HBOT) for chronic radiation cystitis with 5-year follow-up [25], [26] and a randomised study on intravesical sodium hyaluronate with 9-month follow-up [27]. The evidence base was dominated by retrospective studies (*n* = 58) and a small number of prospective cohort studies (*n* = 9), indicating that current practice is supported mainly by observational data. Prostate cancer survivors dominate the studies of urethral stenosis, pubic osteomyelitis, and salvage diversion [19], [75], whereas gynaecologic survivors contributed heavily to ureteral stricture [76], [77], [78], [79], [80], [81] and a component of the fistula series [18], [82]. Follow-up and outcome definitions were heterogeneous. Many studies prioritised technical success, while a smaller group reported pain, urinary bother, health-related quality of life (HRQoL) or treatment burden [28], [75], [83], [84], [85]. Table 3, Table 4, Table 5 summarise the characteristics of individual included studies, grouped by management strategies: bladder-preserving, reconstructive, and salvage management.

### RT characteristics of included cohorts

RT characteristics were extracted from each publication and are summarised in Table 3, Table 4, Table 5. Modality was reported in 39 of 70 publications (56%), the indication for RT (definitive versus adjuvant or salvage) in 31 (44%), total dose or fractionation in 23 (33%), and the interval between RT and the onset of toxicity or its treatment in 49 (70%); 9 publications reported none of these variables. Where dose was reported, it was often available for only part of the cohort, reported medians or protocol doses were 66 to 78 Gy in prostate cohorts [34], [35], [86], [87], [88], [89], [90], with one series also including ultra-hypofractionated schedules of 36.25 Gy [34]; 45 to 50 Gy for EBRT in gynaecologic cohorts [28], [76], with brachytherapy (BT) to a high-risk clinical target volume D90 of 85 to 90 Gy reported in one [76]; and means or medians of 63 to 66 Gy in mixed pelvic HBOT cohorts [31], [36], [37], [74]. Brachytherapy, alone or combined with EBRT, was most prevalent in urethral and salvage cohorts: 43.8% of men undergoing EPA urethroplasty had received brachytherapy and a further 9.5% combined-modality treatment [19], and combined-modality treatment, most often prostatectomy followed by EBRT, accounted for 81% [38] and 80% [39] of two prostate diversion cohorts. Adjuvant or salvage RT after prostatectomy predominated in the AUS and ATOMS literature, accounting for 65 to 100% of patients where reported [40], [41], [42], [87], and was common in fistula and osteomyelitis series [43], [44], [45], [83]. The reported interval from RT to toxicity or treatment varied widely, with medians or means of 7 months to 10 years in the bladder-preserving series and 4 to 19 years in the salvage series (Table 3, Table 5). Prior pelvic surgery was reported principally as prostatectomy preceding RT; reirradiation was reported in four diversion and fistula cohorts, as salvage RT or brachytherapy after RT failure [39], [44], [45] or as palliative EBRT [89]; and concomitant chemotherapy was documented in three ureteral or diversion series [46], [77], [78].

### Synthesised results

Findings are organised primarily by management pathway (bladder-preserving, reconstructive, and salvage), with the relevant urinary syndrome categories addressed within each pathway.

### Bladder-preserving strategies for radiation cystitis, haematuria, and irritative LUTS and urinary bother (Table 3)

Most bladder-preserving studies addressed chronic radiation-induced cystitis (RIC), including its bleeding-predominant subtype, radiation haemorrhagic cystitis (RHC). HBOT was the most extensively evaluated intervention in this category. In the RICH-ART trial, 87 patients were randomised and 79 were analysed, including both men and women and a mixed pelvic cancer population. Mean external-beam dose was 64 Gy, with brachytherapy (BT) (mean 21 and 18.2 Gy) used in 29% (HBOT arm) and 34% (control arm) of patients, and at 6 to 8 months Expanded Prostate Cancer Index Composite (EPIC) urinary total score improved by 17.8 points with HBOT (30–40 sessions, 100% oxygen, breathed at a pressure of 240–250 kPa, for 80–90 min daily) versus 7.7 points with standard care, giving a mean between-group difference of 10.1 points, (95% confidence interval (CI) 2.2 to 18.1, *p* = 0.013) [25]. HBOT also improved EPIC bowel score by 13.2 versus 4.9 points and a higher proportion of patients showed reduced cystoscopic injury on the RTOG/EORTC Late Radiation Morbidity Grading Scheme, 64% versus 18% (*p* = 0.0012). After this randomised endpoint, control patients were offered HBOT; the five-year report is therefore a single-group longitudinal follow-up of the 70 HBOT-treated patients from both arms, not a continued randomised comparison. Mean EPIC urinary total score was 19.1 points above the pre-HBOT baseline at 5 years (95% CI 13.3 to 24.9, *p* < 0.0001; 39 patients with year-5 data), 68.6% had met the responder threshold (improvement of at least 9 points at 6 months after HBOT), and 12.8% received additional HBOT for recurrent symptoms [26].

Observational HBOT cohorts were broadly similar to Oscarsson et al. [25] findings. In a 105-patient cohort with a median follow-up of 64 months, haematuria control was achieved in 92.4%, with recurrence in 24.7% [28]. Earlier treatment after the first haematuria episode was associated with an improved response in both cohorts and lower recurrence in the larger [28], [74], and patients with a complete response had significantly longer haematuria-free survival than those with a partial response, 71.4 versus 36.4 months (*p* < 0.001) [28]. Three of five Late Effects Normal Tissue Subjective, Objective, Management, Analytic (LENT-SOMA) subjective subscores (dysuria, urinary frequency, and haematuria) and the summed score improved significantly, whereas incontinence and decreased stream did not [28]. In a patient-reported follow-up of 54 patients (median 19 months), symptom severity and anxiety scores both improved significantly after HBOT, and general practitioner visits, emergency presentations, and transfusions also declined [30]. Nevertheless, 39% still reported intermittent haematuria and 26% required further treatment. A retrospective two-site comparison found that HBOT delivered at 2.5 rather than 2.0 atm was associated with lower 1-year haematuria recurrence (19.5% vs 38.5%, *p* = 0.018), with more adverse events in one of three analyses [29]; smaller series report broadly consistent complete or partial haematuria resolution rates of 67% to 90% [36], [47]. A 71-patient cohort reported overall success of 64.8% [31]; smaller series reported complete haematuria resolution in 85% of 20 patients at a mean of 24.6 months [37]. In two retrospective cohorts, anticoagulants or antiplatelet therapy was independently associated with recurrence (HR 1.8) [32] or with repeat fulguration (OR 2.8) [34], as was a longer interval from RT to first fulguration (OR 1.8) [34]. In an 80-patient single-centre series of haemorrhagic cystitis after prostate RT, 55% were managed with hydration or temporary catheterisation alone, whereas 45% required hospitalisation; transurethral fulguration was performed in 61% of admitted patients, transfusion in 38.9%, and cystectomy in five, all of whom experienced postoperative complications, and antiplatelet or anticoagulant therapy (HR 3.30) and higher bladder V70 after salvage RT (cut-off 29%) independently predicted admission [33].

Beyond HBOT, the largest body of bladder-preserving evidence comes from intravesical glycosaminoglycan-based therapies. Intravesical sodium hyaluronate significantly improved haematuria and pain from 3 months onward, with QoL domains reaching significant improvement by 6 months, compared with saline in 58 analysed patients [27]. This trend is supported by two prospective cohort studies of 30 patients each: intravesical hyaluronic acid plus chondroitin sulphate produced significant improvement in LUTS and symptom-related bother [48], while weekly sodium hyaluronate instillation produced significant reductions in bladder pain, haematuria, urinary frequency, and the effect of urgency on QoL [49]. A more recent retrospective series (*n* = 30) of Adelmidrol plus sodium hyaluronate reported a significant reduction in pain, with additional improvement in gross haematuria and urgency [50].

Other bladder-preserving haemostatic approaches are supported primarily by retrospective data and appear more useful for selected or rescue settings than as clearly durable therapies. GreenLight laser photocoagulation prevented recurrent haematuria requiring subsequent surgery in 82.7% of patients at a median follow-up of 30 months; however, disabling overactive bladder symptoms developed in 31% of patients, and two patients subsequently required cystectomy [51]. 980-nm diode laser coagulation achieved complete resolution of haematuria in 76.2% of women after a single session, with a 4.8% eventual cystectomy rate [52]. Fibrin glue and formalin achieved rapid haemostatic control in selected cases of severe haemorrhagic cystitis, but these findings derive from single-arm cohorts of 20 and 8 patients respectively and should be interpreted cautiously [53], [54]. Two small single-arm series evaluated regenerative approaches. Intravesical allogeneic platelet lysate, given as 24 instillations over 3 months to 9 men after failed electrocoagulation, achieved complete resolution of haematuria in all patients at 3 months, with residual low-intensity urinary symptoms in up to three patients resolving subsequently, and with cystoscopic and histological normalisation of the mucosa and no recurrence at a median of 56 months [55]. Cystoscopic application of RADA16 self-assembling peptide in 15 men reduced haematuria in 93% at 6 weeks, although Clavien Grade ≥ 3 complications occurred in 27% within 90 days, including repeat application or diathermy and one salvage cystectomy, at a median follow-up of 4 months [56].

For irritative LUTS rather than haematuria, intradetrusor botulinum toxin A significantly reduced daily micturition frequency, nocturia, and pad use in a retrospective series of 28 patients, with these reductions retained in gender-stratified analysis apart from pad use in men [57]. Among dose subgroups, reductions in frequency and nocturia, but not pad use, were significant only at 200 U, whereas International Consultation on Incontinence Questionnaire (ICIQ) Lower Urinary Tract Symptoms quality of life module (ICIQ-LUTSqol) scores improved significantly irrespective of gender or botulinum toxin A dose in the 18 patients with questionnaire data (dose subgroups of 4 to 9 patients). Taken together, these studies suggest that several bladder-preserving treatments can provide symptom control or haemostasis in selected patients, with HBOT remaining the only modality supported by randomised evidence with long-term follow-up.

### Reconstructive strategies for urethral stricture, stress urinary incontinence, and ureteral stricture (Table 4)

#### Urethral stricture and outlet stenosis

Radiation-associated urethral and outlet stenosis were managed predominantly with surgical reconstruction. All cohorts were male, reflecting the anatomical predominance of this side effect in men treated for prostate cancer.

The strongest evidence comes from the two largest multi-institutional reconstructive cohorts. Voelzke et al. reported outcomes of excision and primary anastomosis (EPA) urethroplasty in 137 prostate patients (BT = 43.8%; external beam RT (EBRT) = 38%; combined BT&EBRT = 9.5%; other single-modality RT = 8.7%) achieving instrumentation-free voiding in 86.9% of men at a mean follow-up of 32.3 months [19]. Mean stenosis length was 2.3 cm and mean time to failure was 16.4 months. The functional cost was substantial; postoperative stress incontinence requiring more than one pad daily occurred in 32.1% of patients, 21.9% later underwent artificial urinary sphincter (AUS) placement, and 16.7% of cuffs eroded. Greater stenosis length and increasing age remained independently associated with recurrence on multivariable analysis, whereas combined-modality RT was associated on univariable analysis only [19]. Policastro et al. assessed dorsal onlay buccal mucosal graft urethroplasty in a multi-institutional retrospective series of 79 men [58]. EBRT alone was used in 45.6%, with the remainder receiving BT, EBRT with BT, or prostatectomy with EBRT. Anatomical success, defined as absence of recurrence, was achieved in 82.3% of patients at a median of 21 months. Adverse functional outcomes included de novo stress incontinence in 8.1% of previously continent men. Patient-reported outcomes were favourable with a median International Prostate Symptom Score (IPSS) improved from 21 to 6, and 91.9% of the 37 patients who completed the Global Response Assessment reported Grade + 2 or better, indicating significant symptomatic improvement.

Conservative management was assessed in a two-institution retrospective cohort of 91 elderly men with significant comorbidities (median age 75.4 years), in whom dilation, direct-vision internal urethrotomy, or intermittent catheterisation avoided reconstruction or permanent catheterisation in 80% of patients [59]. Urinary tract infection occurred in 34% and urinary retention in 15% of these patients, supporting a role for non-surgical management in carefully selected patients unfit for reconstruction. Smith and Han reported a single-surgeon series of 16 men with post-radiation prostatic urethral stenosis managed predominantly endoscopically at a mean follow-up of 2.6 years: transurethral resection required further intervention in 48% of cases and dilation in 90%, whereas neither of two urethroplasties recurred; incontinence affected 10 of 16 patients, 5 underwent AUS placement, 3 required chronic catheterisation, and 1 developed a urosymphyseal fistula requiring diversion [60].

Smaller series reported broadly consistent findings. Fuchs et al. reported a recurrence rate of 23.6% after EPA in 72 men at a median of 33.5 months, with stress incontinence remaining common at 34.7% [61], consistent with an earlier series by Rourke et al. reporting 85.7% success after EPA or tissue transfer in 35 men [62]. Barnard et al. reported 91.3% long-term anatomical success and equivalent patient satisfaction at a mean of 73.1 months in 23 men, despite sexual side effects, 95.7% stating they would choose surgery again [63]. Sterling et al. reported seven recurrences and no de novo incontinence in 45 men after dorsal onlay graft urethroplasty at a median of 21 months, with improvements in symptoms and QoL [86]. Early data for the Optilume drug-coated balloon from a multi-institutional retrospective series of 37 men [64] and a series of 30 men [65], showed freedom from repeat intervention of 81.1% and recurrence-free clinical success of 80%, respectively, though follow-up was short in both, and 20% of previously continent men developed new stress incontinence in the latter cohort [65].

Across this literature, reconstruction can restore patency in carefully selected patients, but the consistent finding across series of varying size and design is that continence outcomes, later sphincter surgery requirements, and patient-reported urinary function are as clinically relevant as anatomical patency itself, a pattern likely reflecting the reduced tissue quality of the irradiated field rather than surgical technique alone.

#### Stress urinary incontinence

Post-radiation stress urinary incontinence with reconstructive management was addressed by eight included studies, evaluating artificial urinary sphincter (AUS), adjustable trans-obturator systems, or sling-based continence surgery. In a prospective pilot study of 23 men with severe post-radiation incontinence, AUS implantation with an autologous rectus fascial wrap achieved complete continence in 17 of 22 men with a device in situ (77.3%), with a prosthetic erosion rate of 4.3% at a median of 32 months [42]. Comparative data tend to favour transcorporal cuff placement over standard placement although the evidence is inconsistent. A 45-man retrospective comparison found major complications in 14.8% versus 56%, respectively (*p* = 0.01) [87], whereas a larger 85-man cohort found no significant difference in device survival (removal 36.4% vs 55.8%; HR 0.717, *p* = 0.35) [66]. Across a separate 99-man cohort, time since RT itself did not predict AUS failure, whereas larger cuff size was protective [88]. In a further series of 72 men, device revision or explantation occurred in 50% at a median follow-up of 57.5 months, with no significant difference across 2-, 3-, 4-, or 5-year intervals between RT and implantation [67]. Where stricture of the bladder neck or membranous urethra and incontinence coexist post RT (devastated bladder outlet), revision was more frequent than in RT patients without prior desobstruction (43.1% vs 26.3%, *p* = 0.05) [40]. The adjustable trans-obturator male system offers an alternative in men with residual sphincter function, achieving dryness in 42.6% of 223 men after combined prostatectomy and RT, with baseline incontinence severity the dominant predictor of success [41]. In a study addressing female stress urinary incontinence after pelvic RT, a retrospective series of 26 women treated with mid-urethral sling for incontinence reported 80.8% treatment success at 1 year, with deliberate sling tension, rather than the tension-free principle used in non-irradiated patients, identified as important to surgical success [68]. These data suggest that continence reconstruction is feasible in both sexes, that device and technique selection meaningfully influence outcomes, and that tissue quality and anatomical factors at the point of surgery appear more relevant than time elapsed since RT.

#### Ureteral stricture

Radiation-induced ureteral strictures were managed according to location, length, and complexity. Carilli et al. reported outcomes in 53 patients across multiple institutions, of whom 41.5% had cervical cancer and 22.6% had prostate cancer, all having received EBRT at a median dose of 48 Gy [77]. Management was stratified by stricture complexity: endourology achieved an initial success rate of 74% but only 59% overall success at a median of 12 months, while robotic reconstruction achieved 96% success. Median estimated glomerular filtration rate (eGFR) was unchanged at discharge (71.2 vs 67.7 mL/min, *p* = 0.2) and had improved at a median of 12 months (67.7 to 77.0 mL/min, *p* = 0.007), regardless of treatment strategy. Concomitant chemotherapy independently predicted stricture recurrence (OR 7.8, 95% CI 1.3–49.0, *p* = 0.03). Robotic reconstruction also demonstrated a success rate of 88.2% (30 of 34 evaluable ureteral units; one perioperative death excluded) using tailored techniques in a separate series [69].

Major reconstruction was often required in female gynaecologic cohorts. In a 35-female patient cohort, 82.9% had bilateral disease, and robotic ileal ureter reconstruction achieved 97.1% clinical success at a median of 20 months [79]. Serum creatinine improved from 103.8 to 93.4 μmol/l and eGFR from 66.3 to 77.8 mL/ min/1.73 m^2^ (both *p* < 0.01), with no Clavien Grade ≥ 3 complications. In a separate female (*n* = 18) bilateral ileal ureter substitution study, success was achieved in 94.4% of patients at a median of 24.1 months [76]. A minimally invasive alternative, the thermo-expandable metallic stent, achieved patency rates of 92.4%, 77.4%, and 67.7% at 8, 12, and 29 months, respectively, in a cohort of 27 women, with significant improvement in hydronephrosis (*p* < 0.001) and glomerular filtration rate (*p* = 0.016) at 3 months, whereas the fall in creatinine did not reach significance (*p* = 0.059) [78]. More limited non-bowel reconstructive data also exist; in an 11-patient series of radiation-related pelvic retroperitoneal fibrosis, ureterolysis, ureterotomy, and omental sleeve wrap restored patency in 9 patients [70]. Thomas et al. reported outcomes of enterocystoplasty, often with concomitant ureteric reimplantation, in 36 patients with radiation-related bladder and upper tract injury. Outcomes were more favourable when augmentation was performed concurrently with extirpative surgery; delayed augmentation was associated with catheter dependence, fistula formation, and subsequent cystectomy in a substantial minority of patients, with follow-up extending to a median of 13 years in the extirpative group [71].

The largest ureteral cohort was reported by Polyakov et al., in whom 192 women with distal strictures after RT for gynaecologic cancer underwent extravesical ureteroneocystostomy for defects shorter than 4 cm (*n* = 49) or Boari flap reconstruction with (*n* = 45) or without (*n* = 98) antireflux protection for defects of 4 to 15 cm. Patency was restored in 96%, 95.5%, and 100% of patients respectively at a median follow-up of 38.2 months, recurrences were salvaged by reoperation, and renal function was preserved in all groups; without antireflux protection, clinically significant reflux occurred in 5.1% and pyelonephritis in 18.4% of patients, compared with 6.7% pyelonephritis with antireflux protection (differences not statistically significant) [80]. Huang et al. reported bilateral ileal ureter replacement combined with ileocystoplasty in 7 women with long-segment strictures and bladder contracture; no Clavien Grade ≥ 3 complications occurred, creatinine remained stable, and bladder capacity increased from 99 to 249 mL over 3 to 28 months of follow-up [81].

Across these series, reconstruction preserved or improved renal function in most patients [76], [77], [79], [80], [81], although renal function deteriorated in 13% of a mixed reconstructive cohort despite surgery [18]. Most series were retrospective, conducted in specialist centres, had relatively short follow-up, and, with one exception [80], were small, collectively limiting the generalisability of reported success rates.

### Salvage/End-stage management strategies for fistula, pubic osteomyelitis and end-stage urinary injury (Table 5)

Seventeen studies addressed fistula, pubic osteomyelitis, and end-stage urinary injury. Across all syndromes, high perioperative morbidity was a consistent finding; however, definitive surgery was also associated in most series with reductions in pain, treatment burden, and improvements in patient-reported outcomes.

In women with vesicovaginal fistula after pelvic RT, closure was achieved in 25% of the cohort (5 of 8 attempted repairs), with 70% requiring diversion [82]. For pubic osteomyelitis and urosymphyseal disease, definitive surgery consistently reduced pain across series of varying size and approach, and opioid use where it was measured [43], [44], [45], [83]. In 18 patients undergoing cystectomy, diversion, and pubic symphysis resection, Medical Outcomes Study 12-Item Short Form Health Survey (SF-12) physical composite score improved significantly from 30.6 to 45.5 (*p* = 0.005; *n* = 11 with paired surveys), morphine equivalent use fell significantly from a median of 119.4 to 30.5, and narcotic prescription count decreased from 4.5 to 1.0 (*n* = 16) [83]. Conservative catheter-based care for urosymphyseal fistula carried substantial infection-related mortality, and diversion without cystectomy was associated with more recurrent osteomyelitis than complete extirpation [89].

Urinary diversion with or without cystectomy produced consistent reductions in treatment burden and patient-reported improvements across the end-stage diversion series. Tachibana et al. reported that diversion significantly reduced mean annual procedures from 3.0 to 0.2, hospitalisations from 2.3 to 0.6 among those hospitalised before surgery, and transfused blood units from 7.8 to 0.8 in 45 patients with refractory radiation cystitis [85]. HRQoL assessed after cystectomy and diversion in a 29-patient cohort, measured with a modified SF-36 version 2 (SF-36v2), improved significantly among survey responders, in pain, general health, and the cumulative mental health domain, with 94.7% stating they would have undergone surgery sooner [75]. In a prospective 55-patient diversion cohort, Patient-Reported Outcomes Measurement Information System (PROMIS) physical and mental health scores significantly improved by 7.0 and 7.1 points, respectively, at early follow-up, Genitourinary Pain Index (GUPI) total score significantly improved from 21 preoperatively to 7 at early and 5 at late follow-up (mean 3.8 years), and decision regret remained low at 12.6 at early and 10.4 at late follow-up [84].

Perioperative morbidity was substantial, with Clavien Grade ≥ 3a complications in 28% to 35% after prostate RT diversion, robotic salvage cystectomy, and supratrigonal cystectomy for refractory haemorrhagic cystitis [38], [72], [90]. Any-grade complications occurred within 90 days in 79.4% of women diverted after gynaecologic RT, with high-grade complications in 26.5% [46]. The presence of fistulas outside of the urinary tract was associated, on unadjusted comparison, with higher operative complexity and more Clavien Grade ≥ 3 complications than localised radiation urinary tract injury [39]. Salvage diversion after failed outlet reconstruction remained acceptable to patients, with 81.3% stating they would undergo diversion again [73]. A 13-patient diversion cohort found pain and urinary tract infection improved in 7 of 10 questionnaire respondents, and 8 of the 10 would choose surgery again and sooner [91].

Across the salvage literature, definitive surgery reduced pain, opioid use, admissions, transfusions, and procedural burden in the majority of patients, but perioperative morbidity was high and should be discussed explicitly with patients before intervention.

## Discussion

This review maps the management of late urinary side effects after pelvic RT across 69 studies of previously irradiated patients organised into seven urinary syndrome categories (radiation cystitis and haematuria, irritative LUTS, stress urinary incontinence, urethral stricture, ureteral stricture, fistula/pubic osteomyelitis and end-stage urinary injury) and three complementary management pathways: bladder-preserving, reconstruction, and salvage therapy. Across these syndromes, the literature consistently suggests that treatment choice should be matched to tissue quality, anatomic severity, and overall treatment burden rather than to a simple escalation ladder. This framework improves interpretability by applying explicit criteria to a heterogeneous literature and organising evidence according to the clinical problem and treatment intent most relevant to decision-making. Table 2 maps the interventions reported in the included studies by syndrome category, with factors associated with escalation, limitations, and evidence base for each; it is an evidence map of this restricted literature, not a complete clinical algorithm.

**Table 2:** Evidence map of reported management approaches for late urinary side effects after pelvic radiation therapy

**Management strategy**	Bladder-preserving strategies aim to retain the native bladder; control symptoms or bleeding without major reconstruction or diversion	Reconstructive strategies aim to restore urinary tract patency, continuity, storage, or drainage	Salvage strategies aim to control severe, refractory, or destructive disease, typically via urinary diversion with or without cystectomy or other extirpative surgery


	Syndrome	Interventions reported in the literature	Factors associated with escalation in the mapped literature	Reported limitations and risks	Evidence base
Bladder-Preserving Strategies	**Radiation cystitis and haematuria**	• HBOT for chronic established cystitis supported [25], [26], [29] • Intravesical formalin, fibrin glue, GreenLight or diode laser, glycosaminoglycan instillations, or regenerative agents (platelet lysate, RADA16 peptide), reported for haematuria, its recurrence, or associated symptoms [27], [35], [49], [50], [51], [52], [53], [54], [55], [56]	• Persistent or recurrent bleeding • Transfusion dependence • Repeated admissions • Contracted bladder capacity	•HBOT evidence derives from cohorts with chronic symptoms; the randomised trial excluded patients with recent transfusion-dependent bleeding, bladder capacity below 100 mL, or fistula [25] • HBOT requires 30 to 40 daily sessions and specialist facility access. Haemostatic control may not be durable • Formalin risks contracture at higher concentrations • Antithrombotic therapy is associated with recurrence or repeat intervention [32], [34]; modification requires individualised assessment	1 RCT for HBOT; 1 RCT for sodium hyaluronate; a retrospective comparison associated 2.5 ATA with lower 1-y recurrence than 2.0 ATA [29]; remaining evidence is predominantly retrospective cohorts and case series [28], [30], [31], [32], [33], [34], [36], [37], [47], [74]
	**Irritative LUTS and urinary bother**	• Symptom-directed intravesical therapy (hyaluronic acid-based instillation) [27], [48], [49], [50] Intradetrusor botulinum toxin A injection, an emerging option for radiogenic LUTS [57]	• Worsening capacity • Refractory pain • Recurrent infection • Emerging incontinence suggesting more advanced tissue injury	• Symptom burden may reflect progressive tissue injury, not isolated mucosal irritation	Small prospective/retrospective cohorts only; one retrospective series (*n* = 28) reported reduced frequency, nocturia, and pad use after BTX-A; among dose subgroups, reductions in frequency and nocturia were significant only at 200 U, whereas ICIQ-LUTSqol improved across sex and dose subgroups (*n* = 18) [57]
Reconstructive strategies	**Stress urinary incontinence**	• Assessment distinguishing sphincteric versus outlet mechanism • ATOMS (adjustable trans-obturator male system) in men with residual sphincter function [41] • Continence reconstruction: AUS in men [40], [42], [66], [67], [87], [88], mid-urethral sling in women [68]	• Severe leakage • Prior failed continence surgery • Devastated bladder outlet (concurrent stenosis and incontinence) • Major quality-of-life burden	• Continence gains may be offset by erosion, prosthetic failure, sling-related dysuria, or later reintervention • Devastated outlet (concurrent stricture) carries higher revision rates [40]	Predominantly retrospective cohorts, several multicentre; no RCT evidence. AUS feasible in irradiated men, larger cuff associated with lower device failure [88]; Evidence on transcorporal versus standard cuff placement is inconsistent, one comparative series favouring transcorporal placement [87] and a larger series finding no difference in device survival [66]. ATOMS effective post-prostatectomy/RT, dryness dependent mainly on baseline severity [41]; sling feasible in irradiated women [68]
	**Urethral stricture**	• EPA urethroplasty reported for bulbomembranous stenosis [19], [61], [62], [63] • Buccal mucosal graft urethroplasty for longer or complex strictures [58], [62], [86] • Drug-coated balloon dilation where reconstruction is not feasible [64], [65] • Endoscopic management, dilation, or intermittent catheterisation in patients unfit for reconstruction [59], [60]	• Repeat recurrences after endoscopic treatment • Worsening continence • Severe fibrosis precluding further instrumentation	• Repeated endoscopic treatment worsens tissue damage • Stress incontinence (32 to 35%) and later AUS placement (0 to 22%) are common after EPA [19], [61], [63]; de novo incontinence was 0 to 8% after dorsal onlay grafting [58], [86]	Retrospective cohort series; patency 76–91% for EPA [19], [61], [62], [63] and 82 to 84% after dorsal onlay buccal mucosal graft urethroplastyurethroplasty [58], [86]. Early balloon data are promising but follow-up is short [64], [65].
	**Ureteral stricture or upper tract obstruction**	• Renal function preservation reported as a primary outcome [18], [76], [77], [78], [79], [80], [81] • Endourology for short proximal strictures [77] • Robotic or open reconstruction (ureteral reimplantation, Boari flap, ileal ureter substitution, augmentation) for longer or complex disease [69], [71], [76], [77], [79], [80], [81] • Thermo-expandable metallic stents, a minimally invasive option, particularly after gynaecologic RT [78] • Ureterolysis with omental wrap for radiation-related retroperitoneal fibrosis [70]	• Bilateral disease • Long-segment stricture • Recurrent obstruction • Declining renal function	• Complexity is frequently underestimated in irradiated fields • Stricture length over 2 cm and concomitant chemotherapy (the latter on multivariable analysis) predict higher recurrence risk [77] • Expert-centre bias limits generalisability	Retrospective cohorts from expert centres; robotic reconstruction success 88–97%; open reimplantation or Boari flap patency 96–100% in a 192-patient series [80]; endourology success lower for complex disease; metallic stent patency 67.7–92.4% at 8–29 months [78].
Salvage strategies	**Fistula and pubic osteomyelitis**	• Multidisciplinary assessment with cross-sectional imaging (MRI) [20], [89] • Surgical repair with flap interposition for fistula [43], [44], [45], [82], [89] • Debridement +/−pubectomy for osteomyelitis [43], [44], [45], [83], [89] • Diversion or cystectomy if repair not feasible [20], [45], [82], [83], [89]	• Persistent sepsis • Severe or refractory pain • Ongoing urinary leak • Non-salvageable outlet	• High perioperative morbidity [45], [83] • Conservative management rarely succeeds [44], [89]	Small-to-moderate case series; high surgical success for pain/infection control. Closure was achieved in 25% and diversion required in 70% of irradiated vesicovaginal fistulae [82]
	**End-stage urinary injury (refractory bleeding or devastated outlet)**	• Urinary diversion with or without cystectomy, including robotic-assisted approaches [38], [39], [46], [72], [73], [75], [84], [85], [90], [91] • Multidisciplinary planning reported across series • Repeated prior endoscopic or reconstructive intervention common before diversion [38], [39], [73] • Pedicled flap interposition reported [38], [39], [73]	• Failure of conservative or reconstructive options • Rising treatment burden (admissions, transfusions, procedures) • Patient preference after informed discussion	• Major perioperative complication rate is significant [38], [46], [72], [90] • Patient-reported benefit supports timely referral and shared decision-making; no included study compared earlier with delayed surgery	Predominantly retrospective cohorts; diversion reduces admissions, transfusions, and pain burden, with measurable PROM gains. Most questionnaire respondents would choose surgery again, sooner [91].

This table is an evidence map of the interventions reported in the studies included in this review, which was restricted to cohorts of previously irradiated patients published from 2015. It is not a complete clinical algorithm, and the absence of an intervention indicates only that no eligible study was identified. The seven syndrome categories are separate clinical conditions, grouped by management pathway and not ordered by severity. Before haematuria is attributed to radiation cystitis, infection, recurrent disease, secondary malignancy, and other urinary sources should be excluded.

Abbreviations: RT, radiation therapy; HBOT, hyperbaric oxygen therapy; LUTS, lower urinary tract symptoms; ATA, atmospheres absolute; BTX-A, botulinum toxin A; AUS, artificial urinary sphincter; ATOMS, adjustable trans-obturator male system; EPA, excision and primary anastomosis; RCT, randomised controlled trial; PROM, patient-reported outcome measure; MRI, magnetic resonance imaging. NR, not reported; 3D-CRT, three-dimensional conformal radiation therapy; IMRT, intensity-modulated radiation therapy; SBRT, stereotactic body radiation therapy; HDR, high dose rate; LDR, low dose rate; fr, fractions; mo, months; y, years.

HBOT has the clearest evidence base in chronic radiation cystitis, where it stands apart from the rest of the bladder-preserving literature. It is the only modality supported by randomised evidence and long-term follow-up, and it is also the best-supported conservative therapy from a patient-reported outcome perspective [25], [26]. By contrast, formalin, laser coagulation, intravesical barrier-restoring therapies, and emerging regenerative agents show benefit in selected settings but are supported mainly by small retrospective or single-arm cohorts with heterogeneous definitions of success [35], [51], [52], [54], [55], [56]. These treatments still have value, but should be understood as addressing specific symptoms or providing temporary control, rather than as alternatives supported by evidence comparable to that for HBOT. This pattern is most evident across the ablative and instillation literature: rapid haemostatic control is achievable, yet disabling storage symptoms, recurrent bleeding, and progression to cystectomy remain real risks [51], [52], [54].

The reconstructive literature supports surgery for carefully selected patients, but it also makes clear that technical success alone is insufficient. In urethral disease, excellent patency can coexist with substantial stress incontinence and later AUS placement [19], [58], and with persisting storage symptoms or urinary bother [60], [62], [63]. This is where patient-reported outcomes become especially important. A urethra that remains open may still leave the patient with major leakage or impaired QoL, whereas a technically imperfect outcome may still be perceived as worthwhile if pain, obstruction, or catheter dependence are relieved. The same principle applies to upper-tract reconstruction. Ureteral success is best understood not only in terms of stricture recurrence but also in terms of renal preservation, avoidance of chronic tubes or stents, the feasibility of major bowel-based reconstruction in irradiated fields [76], [79], [81], and the durable patency of reimplantation or Boari flap reconstruction in the largest reported series [77], [80].

Radiation dose and modality were inconsistently reported across the included literature, though this has improved in some recently published series [25], [77]. Only a minority of publications reported total dose, and fewer than half reported the indication for RT, which limits any synthesis of dose–toxicity relationships. Once a side effect such as stricture, fistula, or haemorrhagic cystitis is established, management is determined principally by anatomy, severity, and tissue quality rather than by the nominal dose that caused it. Prior modality and cumulative exposure nevertheless remain clinically relevant as markers of tissue-injury burden, and the included studies provide within-study evidence of this: greater stenosis length independently predicted, and more than one radiation modality was associated on univariable analysis with, recurrence after EPA urethroplasty [19], failure of conservative management was highest after combined EBRT and brachytherapy [59], higher bladder V70 after salvage RT predicted hospitalisation for haemorrhagic cystitis [33], adjuvant RT was associated with failure of glycosaminoglycan instillation [35], and concomitant chemotherapy predicted ureteral stricture recurrence [77]. Brachytherapy and combined-modality treatment feature prominently in the severe urethral and salvage cohorts [19], [38], [39], consistent with the substantially higher urethral and bladder-base doses these approaches deliver relative to EBRT alone, and prostatectomy followed by adjuvant or salvage RT is common in the incontinence and fistula literature [40], [41], [44], [45], [87]. Taken together, these findings support organising management by syndrome and severity, while treating prior modality, dose, sequencing with surgery, and interval since RT as modifiers of reconstructive complexity, healing capacity, and complication risk that should be documented and considered when selecting and timing intervention. Apparent sex differences largely reflect syndrome distribution rather than differential treatment response. Male-dominant cohorts predominate in urethral and prostate-related pathology [19], [38], [83] and female-dominant cohorts in ureteral and gynaecologic reconstruction [46], [76], [79], [80], [81], [82], with sex-specific outcome reporting remaining limited even in mixed cohorts.

Patient-reported outcomes are among the most important advances in recent literature. Older studies often emphasized patency, haemostasis, or perioperative morbidity alone. More recent work shows that these endpoints capture only part of the clinical reality for patients. HBOT significantly improves EPIC urinary scores [25], [26]. Urethroplasty studies reporting PROMs or IPSS show that symptom burden and continence outcomes can diverge from anatomic success [58], [62], [63]. Salvage surgery, despite its morbidity, now has some of the strongest patient-centred evidence in the field: significant SF-36 improvements in pain, general health, and mental health after cystectomy [75], significant PROMIS and GUPI gains alongside low decision regret after diversion [84], and significant improvement in physical function with reduced opioid burden after surgery for pubovesical fistula and osteomyelitis [83]. These findings suggest that future studies should treat patient-reported outcomes as core rather than optional outcomes.

The salvage literature carries a final clinical implication. Diversion or cystectomy should not be regarded solely as treatment failure. In refractory bleeding, fistula, pubic osteomyelitis, and devastated outlet, these operations often provide the most substantial reduction in pain [84] and in transfusions, hospital admissions, and procedures [85]. Perioperative morbidity remains substantial, including in female diversion series after gynaecologic RT [46] and in older prostate salvage cohorts with mean or median ages of 71 to 80 years [38], [39], [90]. Nonetheless, available patient-reported data indicate that many patients judge these operations worthwhile once durable symptom control is achieved. This reframes the clinical question from whether salvage surgery is too radical to whether repeated low-yield interventions become more harmful than definitive reconstruction or diversion [20], [38], [39], [73]. The included studies did not compare earlier with delayed surgery, so these data support timely referral and shared decision-making and do not establish an optimal timing for surgery. These findings also indicate an opportunity for radiation oncology services to develop structured referral pathways for late urinary side effects, in which syndrome, severity, and tissue quality inform the timing of reconstructive or salvage assessment.

The main limitations of this review are the retrospective nature of most included studies, heterogeneity in endpoints, likely referral bias toward expert centres, and inconsistent reporting of radiation details. Title and abstract screening was performed by a single reviewer, which may have introduced selection error at that stage; full-text eligibility was assessed by two reviewers with strong agreement (κ = 0.80), and data extraction was verified for a 20% sample. Risk of bias was assessed for the randomised trials only, by a single reviewer; the non-randomised studies were not formally appraised. Patient-reported outcomes remain unevenly distributed across the literature, and some syndromes remain represented mainly by small series. The 2015 publication cut-off bounds the literature by date of report rather than by radiation era: because late injury can present years after treatment, the included cohorts contain patients irradiated with older techniques, and earlier reports of approaches that remain in use may have been excluded. This review restricts inclusion to cohorts in which all patients had been irradiated, which improves clinical specificity but may omit some mixed-cohort comparator data. All cohorts had received pelvic radiation therapy, but most patients had also undergone surgery, chemotherapy, or androgen deprivation, reflecting standard multimodal management. Radiation exposure was therefore established, whereas attribution of injury to radiation rather than to other pelvic treatment rests on the original authors' judgement and cannot be verified in observational data. Late toxicity was defined as arising more than 90 days after RT, but timing was not a screening criterion: the interval from RT was not reported for every cohort, and in some the reported range included patients presenting within 90 days, so a minority of patients with acute or subacute injury may be included.

## Conclusions

Late urinary side effects after pelvic RT span a wide management spectrum, from bladder-preserving therapy to complex reconstruction and extirpative salvage surgery. Management should be matched to the syndrome, tissue quality, and treatment burden rather than to a fixed escalation ladder, with recognition of when repeated low-yield intervention should prompt referral for reconstructive or salvage assessment. Prior RT modality, dose, sequencing with surgery, and interval since treatment do not dictate the choice of intervention once injury is established, but they mark tissue-injury burden, modify reconstructive complexity and complication risk, and should be documented when selecting and timing treatment. Patient-reported outcomes are central to defining treatment success in irradiated tissue, and salvage surgery, despite its morbidity, was associated with substantial patient-reported benefit in the included series. No included study compared early with delayed surgery; the evidence therefore supports timely specialist referral and shared decision-making but does not establish the optimal timing of surgery. Future studies should adopt prospective designs and standardise reporting of radiation dose, and patient-reported outcomes to refine this decision-making framework. More consistent reporting of RT characteristics would allow radiation exposure and management outcomes to be examined directly.

## CRediT authorship contribution statement

**Laura Mullaney:** Conceptualization, Methodology, Investigation, Data curation, Formal analysis, Writing – original draft, Writing – review & editing, Project administration, Supervision. **Salma Bennani:** Conceptualization, Methodology, Investigation, Data curation, Writing – review & editing.

## Declaration of generative AI and AI-assisted technologies in the writing process

During the preparation of this work, the authors used Claude to improve the final draft's readability and reduce its word count. The authors reviewed and edited the output as needed and take full responsibility for the content of the published article.

## Funding

This research did not receive any specific grant from funding agencies in the public, commercial, or not-for-profit sectors.

## Declaration of competing interest

The authors declare that they have no known competing financial interests or personal relationships that could have appeared to influence the work reported in this paper.

## Data Availability

A charted dataset was created and analysed in this study. The charted data are presented in Table 3, Table 4, Table 5 and the appendices.

## Appendix A Supplementary data

Supplementary material Search Strategy and PRISMA-ScR checklist.
