# Supplementary material for: Management of late urinary side effects after pelvic radiation therapy: a scoping review
Source: Clin Transl Radiat Oncol. 2026 Oct 21;62:101332. doi: 10.1016/j.ctro.2026.101332 (PMC13757291; doi:10.1016/j.ctro.2026.101332)
Supplement: Supplementary file 1 [file mmc1.docx]

# Appendices

### Appendix A

### PubMed Search Strategy

#1 “Radiotherapy”[Mesh] OR radiotherapy*[tiab] OR radiation[tiab] OR irradiat*[tiab] OR brachytherap*[tiab] OR “pelvic radiotherapy”[tiab] OR “pelvic radiation”[tiab]

#2 pelvis[tiab] OR pelvic[tiab] OR prostate[tiab] OR cervical[tiab] OR cervix[tiab] OR gynecolog**[tiab] OR gynaecolog**[tiab] OR endometrial[tiab] OR uterine[tiab] OR rectal[tiab] OR colorectal[tiab] OR anal[tiab] OR bladder[tiab]

#3 “Cystitis”[Mesh] OR “Hematuria”[Mesh] OR “Urinary Incontinence”[Mesh] OR “Urethral Stricture”[Mesh] OR “Ureteral Obstruction”[Mesh] OR “Lower Urinary Tract Symptoms”[tiab] OR LUTS[tiab] OR “radiation cystitis”[tiab] OR “hemorrhagic cystitis”[tiab] OR “haemorrhagic cystitis”[tiab] OR hematuria[tiab] OR haematuria[tiab] OR dysuria[tiab] OR urgency[tiab] OR frequency[tiab] OR incontinence[tiab] OR “stress urinary incontinence”[tiab] OR stricture**[tiab] OR stenosis[tiab] OR urethr**[tiab] OR ureter**[tiab] OR fistula**[tiab] OR osteomyelitis[tiab] OR “pubic bone osteomyelitis”[tiab] OR “urosymphyseal fistula”[tiab]

#4 “Therapeutics”[Mesh] OR treatment[tiab] OR management[tiab] OR intervention*[tiab] OR surgery[tiab] OR surgical[tiab] OR reconstruction[tiab] OR urethroplasty[tiab] OR diversion[tiab] OR cystectomy[tiab] OR “hyperbaric oxygen”[tiab] OR instillation*[tiab] OR embolization[tiab] OR embolisation[tiab] OR fulguration[tiab] OR laser[tiab] OR botulinum[tiab] OR “artificial urinary sphincter”[tiab] OR sling[tiab] OR Optilume[tiab]

#5 #1 AND #2 AND #3 AND #4

#6 #5 AND (“2015/01/01”[Date - Publication] : “2026/04/29”[Date - Publication])

#7 #6 NOT (animals[mh] NOT humans[mh])

### Embase search (Embase.com, Elsevier)

#1: (‘radiotherapy’/exp OR ‘brachytherapy’/exp OR radiotherapy*:ti,ab OR irradiat*:ti,ab OR brachytherap*:ti,ab OR ‘radiation therap*’:ti,ab OR ’external beam radiotherapy*’:ti,ab OR ebrt:ti,ab OR imrt:ti,ab)

#2: (pelvic:ti,ab OR pelvis:ti,ab OR prostate:ti,ab OR cervical:ti,ab OR cervix:ti,ab OR gynecolog**:ti,ab OR gynaecolog**:ti,ab OR endometrial:ti,ab OR uterine:ti,ab OR rectal:ti,ab OR colorectal:ti,ab OR anal:ti,ab OR bladder:ti,ab)

#3: (‘radiation cystitis’/exp OR ‘hemorrhagic cystitis’/exp OR ‘hematuria’/exp OR ‘urinary incontinence’/exp OR ‘urethra stricture’/exp OR ‘ureter obstruction’/exp)

#4: (‘radiation cystitis’:ti,ab OR ‘haemorrhagic cystitis’:ti,ab OR ‘hemorrhagic cystitis’:ti,ab OR hematuria:ti,ab OR haematuria:ti,ab OR dysuria:ti,ab OR urgency:ti,ab OR frequency:ti,ab OR incontinence:ti,ab OR ‘stress urinary incontinence’:ti,ab OR ’lower urinary tract symptom*’:ti,ab OR luts:ti,ab OR stricture*:ti,ab OR stenosis:ti,ab OR urethr*:ti,ab OR ureter*:ti,ab OR fistula*:ti,ab OR osteomyelitis:ti,ab OR ‘urosymphyseal fistula’:ti,ab OR ‘pubic bone osteomyelitis’:ti,ab)

#5: (‘therapy’/exp OR ‘surgery’/exp OR ‘reconstructive surgery’/exp OR ‘urinary diversion’/exp OR ‘cystectomy’/exp OR treatment:ti,ab OR management:ti,ab OR intervention*:ti,ab OR surgery:ti,ab OR surgical:ti,ab OR reconstruction:ti,ab OR urethroplasty:ti,ab OR diversion:ti,ab OR cystectomy:ti,ab OR hyperbaric oxygen:ti,ab OR instillation*:ti,ab OR embolization:ti,ab OR embolisation:ti,ab OR fulguration:ti,ab OR laser:ti,ab OR botulinum:ti,ab OR ‘artificial urinary sphincter’:ti,ab OR sling:ti,ab OR optilume:ti,ab)

#6: #1 AND #2 AND (#3 OR #4) AND #5

#7: #6 AND [humans]/lim AND [2015-2026]/py

Additional filters: Age 18+; Publication type — not abstract only or conference abstract; Human

### Appendix B: Risk of bias in randomised trials (RoB 2)

The two randomised trials were assessed using RoB 2. Non-randomised studies were not formally appraised.

| **Study (Ref)** | **Randomisation process** | **Deviations from intended interventions** | **Missing outcome data** | **Measurement of the outcome** | **Selection of the reported result** | **Overall** |
| --- | --- | --- | --- | --- | --- | --- |
| Oscarsson 2019 (25); five-year follow-up, Oscarsson 2025 (26) | Low | Some concerns | Some concerns | High | Low | High |
| Allameh 2020 (27) | Low | Some concerns | Low | Low | Some concerns | Some concerns |

Footnote: Assessed for the effect of assignment to intervention. The judgement for Oscarsson 2019 covers both the primary report (25) and the five-year follow-up (26), which report the same trial. Risk-of-bias judgements apply to the specified outcome and are not a ranking of overall study quality. The high-risk judgement for Oscarsson 2019 arises from the measurement of a patient-reported outcome in an unblinded trial, a design constraint inherent to hyperbaric oxygen delivered without a sham comparator, rather than from any deficiency in trial conduct or reporting.

### Appendix C: Publications reporting overlapping patient populations

| **Publications** | **Basis** | **Evidence** |
| --- | --- | --- |
| ^a^ Ceballos 2025; Masopeh 2025 | Confirmed by authors | Series share patients (L. Wiegand, personal communication) |
| ^b^ Policastro 2021; Sterling 2024 | Confirmed by authors | 18 patients common to both series; the latter report adds longer follow-up and further patients (D. Nikolavsky, personal communication) |
| ^c^ Malshy 2024 (*World J Urol*); Malshy 2024 (*Urology*) | Confirmed by authors | Shared source population with substantial patient overlap (K. Malshy, personal communication) |
| ^d^ Toia 2019; Toia 2020 | Inferred | Same unit; recruitment 2007–2017 and 2009–2018; shared authorship |

Voelzke 2021 and Bassett 2017 draw on the same research network, and Li 2021, Huang 2024 and Huang 2026 on the same centre, but report mutually exclusive interventions over non-overlapping or minimally overlapping recruitment periods.

Appendix D

**PRISMA-ScR checklist**

Management of late urinary side effects after pelvic radiation therapy: a scoping review. Items condensed from Tricco AC, Lillie E, Zarin W, O'Brien KK, Colquhoun H, Levac D, Moher D, Peters MD, Horsley T, Weeks L, Hempel S. PRISMA extension for scoping reviews (PRISMA-ScR): checklist and explanation. Annals of internal medicine. 2018 Oct 2;169(7):467-73.

| **Section** | **#** | **PRISMA-ScR item** | **Reported in section** | **Page** |
| --- | --- | --- | --- | --- |
| **TITLE** | | | | |
| Title | 1 | Identify the report as a scoping review. | Title | 1 |
| **ABSTRACT** | | | | |
| Structured summary | 2 | Structured summary: background, objectives, eligibility criteria, sources, charting methods, results, conclusions. | Abstract | 2 |
| **INTRODUCTION** | | | | |
| Rationale | 3 | Rationale for the review and for a scoping approach. | Introduction, paragraphs 1 to 4 | 3 |
| Objectives | 4 | Explicit statement of questions and objectives, with key elements (PCC). | Introduction, final paragraph; Methods; Table 1 | 3, 17 |
| **METHODS** | | | | |
| Protocol and registration | 5 | State whether a protocol exists and where it is registered. | Methods, opening paragraph | 3 |
| Eligibility criteria | 6 | Eligibility criteria (years, language, publication status) with rationale. | Methods; Table 1 | 3–4, 17 |
| Information sources | 7 | All information sources, dates of coverage and date of last search. | Search Strategy, Sources, and Screening | 4 |
| Search | 8 | Full search strategy for at least one database, including limits. | Appendix A | S1 |
| Selection of sources of evidence | 9 | Process for screening and eligibility assessment. | Search Strategy, Sources, and Screening | 4 |
| Data charting process | 10 | Charting methods, forms, duplication and verification. | Data Extraction; Appendix C | 5; S4 |
| Data items | 11 | Variables sought, with assumptions and simplifications. | Data Extraction; Classification Framework | 4–5 |
| Critical appraisal of individual sources of evidence | 12 | If done, rationale, methods and use in synthesis. | Risk of Bias Assessment | 5 |
| Synthesis of results | 13 | Methods of handling and summarising charted data. | Data Extraction; Classification Framework | 4–5 |
| **RESULTS** | | | | |
| Selection of sources of evidence | 14 | Numbers screened, assessed and included, with reasons for exclusion; flow diagram. | Results, first paragraph; Figure 1 | 5, 17 |
| Characteristics of sources of evidence | 15 | Characteristics charted for each source, with citations. | Results; Tables 3 to 5 | 6, 22–41 |
| Critical appraisal within sources of evidence | 16 | If done, appraisal data for included sources. | Results, second paragraph; Appendix B | 5; S2 |
| Results of individual sources of evidence | 17 | Charted data for each source relevant to the objectives. | Tables 3 to 5 | 22–41 |
| Synthesis of results | 18 | Charting results summarised against the objectives. | Synthesised Results; Table 2 | 6–13, 18 |
| **DISCUSSION** | | | | |
| Summary of evidence | 19 | Main results, link to objectives, relevance to key groups. | Discussion, paragraphs 1 to 6 | 13–14 |
| Limitations | 20 | Limitations of the scoping review process. | Discussion, limitations paragraph | 15 |
| Conclusions | 21 | General interpretation, implications and next steps. | Conclusions | 15 |
| **FUNDING** | | | | |
| Funding | 22 | Funding of included sources and of the review; role of funders. | Funding statement | 42 |
